# Climate-driven selection may shape mitochondrial genome evolution in Scincidae

**DOI:** 10.1016/j.isci.2026.116789

**Published:** 2026-07-14

**Authors:** Xuxiang Wu, Lemei Zhan, Lingyi Ding, Lining Chen, Runan Zheng, Xingzhou Ma, Jiayong Zhang, Danna Yu

**Affiliations:** 1 College of Life Sciences, Zhejiang Normal University, Jinhua 321004, China; 2 Key Lab of Wildlife Biotechnology, Conservation and Utilization of Zhejiang Province, Zhejiang Normal University, Jinhua 321004, China

**Keywords:** Scincidae, mitochondrial genome, climate-driven selection, positive selection, phylogenetic relationships, divergence time

## Abstract

Skinks (family Scincidae) are one of the most widely distributed lizard groups, and hence they are likely subjected to varying selective pressures and metabolic demands. In this study, we assembled 16 complete mitochondrial genomes from skink species. Phylogenetic relationships and divergence times within Scincidae were reconstructed using these data. Subsequently, 36 distinct skink species were classified into five distinct clusters based on bioclimatic variables. Notably, positively selected sites were detected at amino acid position 93 of the *ND2* gene in the cluster A (*Chalcides ocellatus* and *C. sepsoides*) and at position 267 of the *ND1* gene in the cluster 2 (including members of Scincinae and Sphenomorphinae). Environmental correlation analyses revealed that temperature seasonality and precipitation seasonality exhibited the strongest statistical associations with mitochondrial protein-coding genes, particularly *COX3* and *ND1* genes. These results suggest that the mitochondrial genes of skinks may have been shaped by climate-driven selection.

## Highlights

• 16 complete skink mitogenomes assembled, enabling phylogeny and divergence time estimation
• 36 skink species grouped into five distinct climatic clusters from seven bioclimatic variables
• Positively selected sites detected at *ND2* and *ND1* in climate-defined clusters A and 2
• Temperature seasonality and precipitation seasonality associations with *COX3* and *ND1* evolution

## Graphical abstract

**Figure:**
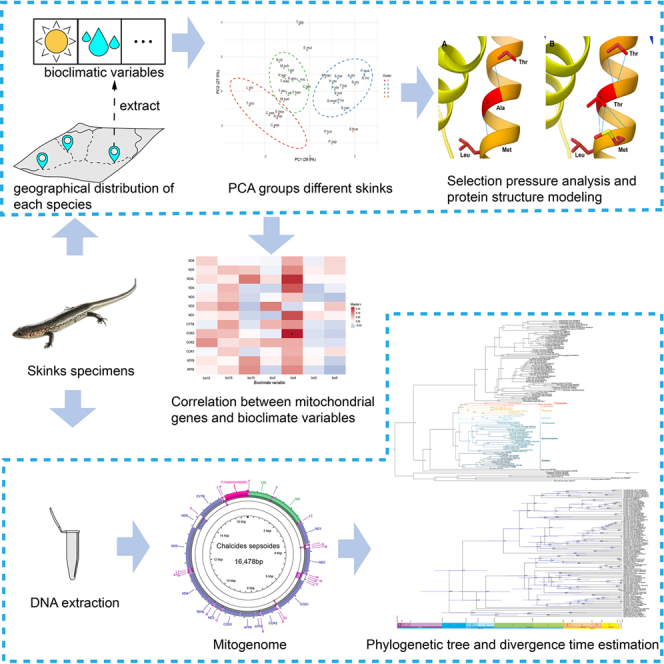

## Teaser

Zoology; Phylogenetics; Evolutionary biology; Phylogeny

## Introduction

The family Scincidae currently comprises 1,993 recognized species (http://www.reptile-database.org, accessed on July 24, 2025). The high numbers reflect substantial species richness and taxonomic diversity.^1^^,^^2^ Skinks are characterized by a broad global distribution, occurring on all continents except Antarctica.^3^ Major centers of skink diversity are located in Southeast Asia, New Guinea, Oceania, Madagascar, and Central Africa.^3^

Despite their widespread distribution and high diversity, the phylogenetic relationships within the family Scincidae remain poorly resolved. Based on cranial morphology, Greer^4^ originally classified Scincidae into four subfamilies: Acontinae, Feylininae, Lygosominae, and Scincinae. However, with the advent of molecular phylogenetic approaches, subsequent studies^5^^,^^6^ have demonstrated that Feylininae is deeply nested within Scincinae, leading to the formal synonymization of Feylininae within Scincinae. The extensive species richness of Scincidae has posed significant challenges for its classification, resulting in overly inclusive higher taxonomic ranks, particularly at the genus level and above, thereby complicating taxonomic revisions and the description of new species. Notably, recent molecular phylogenies have provided robust support for certain previously contentious taxonomic groupings: the validity of Acontinae as a distinct subfamily has been confirmed; intergeneric relationships within Scincinae and Lygosominae remain largely unresolved or inconsistent across studies; and the monophyly of five informal groups within Lygosominae has been substantiated. Based on these findings, Hedges and Conn^7^ proposed a seven-subfamily classification system in 2012. They split the former Lygosominae into five distinct subfamilies: Egerniinae, Eugongylinae, Lygosominae, Mabuyinae, and Sphenomorphinae, while retaining Acontinae and Scincinae as separate subfamilies. Subsequently, in 2014, Hedges^8^ proposed adding two subfamilies, Ristellinae and Ateuchosaurus, bringing the total to nine families. In this study, we follow the skink classification system of Hedges.^8^ Inherent properties of mitochondrial sequences, including their relatively short length, rich gene polymorphism, and absence of recombination, make them well-suited for reconstructing phylogenetic relationships.^9^ To date, no complete mitochondrial genomes for representatives of Lygosominae are publicly available in NCBI (https://www.ncbi.nlm.nih.gov, accessed on July 15, 2025). Therefore, the use of complete mitogenomes to infer the phylogenetic relationships among the six recognized subfamilies of Scincidae (Scincinae, Sphenomorphinae, Egerniinae, Mabuyinae, Lygosominae, and Eugongylinae) remains unexplored. Consequently, reconstructing the phylogeny of major Scincidae lineages using complete mitogenomic data represents a scientifically valuable pursuit.

The widespread distribution of skinks suggests that different species are frequently exposed to divergent environmental pressures, particularly climatic factors.^10^ Temporally variation in temperature and precipitation often serves as a potent selective pressure,^11^^,^^12^ promoting adaptive evolutionary responses that enhance fitness under specific climatic conditions. These adaptations may manifest at multiple biological levels, including behavioral, morphological, physiological, and metabolic traits.^13^^,^^14^^,^^15^ For example, in cold environments at high latitude and elevations, a greater proportion of skink species exhibit viviparity, as the maternal uterus provides superior thermal insulation compared with externally deposited eggs, thereby improving offspring survival under low ambient temperatures.^16^^,^^17^^,^^18^ Morphologically, skinks display extensive variation in body form and size, ranging from terrestrial species with broad heads, robust builds, and adaptations for inhabiting rock crevices (e.g., *Plestiodon chinensis*) to arboreal forms with slender limbs and agile locomotion (e.g., *Dasia vittata*) and to limbless forms characterized by reduced heads and elongated, cylindrical bodies (e.g., species within Acontinae). As ectotherms that depend primarily on external heat sources for thermoregulation,^19^^,^^20^ skinks are highly sensitive to fluctuations in ambient climatic conditions.^21^^,^^22^

Mitochondria play a central role in eukaryotic energy metabolism, generating the majority of cellular ATP^23^^,^^24^ while remaining highly sensitive to fluctuations in external temperature.^25^^,^^26^ As semi-autonomous organelles,^27^^,^^28^ mitochondria possess their own double-stranded, circular DNA molecule. The mitochondrial genome typically ranges from 15 to 20 kb in length and consists of 13 protein-coding genes (PCGs), 2 rRNA genes, 22 tRNA genes, and a control region.^29^^,^^30^ The proteins encoded by these 13 PCGs constitute essential subunits of the respiratory chain complexes.^31^ They mediate electron transfer through the electron transport chain and establish a proton gradient across the inner mitochondrial membrane, thereby driving oxidative phosphorylation (OXPHOS) for ATP production.^32^^,^^33^ Consequently, mitochondrial PCGs serve as effective molecular markers for detecting natural selection.^34^^,^^35^ This is because species inhabiting different climatic zones experience divergent metabolic demands, which may subject mitochondrial PCGs to directional or adaptive selection. For example, selection pressure analyses comparing two *Tetranychus* (Acariformes, Acari) species from contrasting climates have revealed diversifying positive selection in *ATP6* and *ND4*.^36^ Similarly, pronounced altitudinal and precipitation gradients in the Balkans may impose selection pressure on *ND6* in *Lepus europaeus* (Lagomorpha, Mammalia), where one positively selected site was detected.^37^ Comparative mitogenomic analysis of decapods from contrasting environments (equatorial high-temperature and deep-sea low-oxygen habitats) has revealed signatures of positive selection in mitochondrial complex I, III, IV, and V genes, suggesting that local adaptation to temperature and oxygen levels drives adaptive evolution in Achelata (Decapoda, Malacostraca) mitochondrial PCGs.^38^

Accordingly, this study aims to achieve the following objectives: (1) generate multiple mitogenomes from the family Scincidae to reconstruct phylogenetic relationships and estimate divergence times among lineages, (2) quantify selection pressures across different clusters, and (3) evaluate the strength of associations between bioclimatic variables and mitochondrial PCGs.

## Results

### Mitochondrial genome statistics

A total of 16 mitochondrial genomes were obtained. Eight sequences were generated through *de novo* sequencing in this study, including *Chalcides sepsoides* (16,478 bp), *Eutropis multifasciata* (16,880 bp), *Lipinia microcerca* (17,494 bp), *Scincella vandenburghi* (17,169 bp), *Sphenomorphus indicus* (16,830 bp), *Sp. cryptotis* (17,351 bp), *Sp. incognitus* (17,115 bp), and *Sp. maculatus* (17,225 bp). The remaining eight mitogenomes were assembled from raw sequencing reads retrieved from the SRA database, including *Eulamprus heatwolei* (17,116 bp), *Mesoscincus schwartzei* (16,897 bp), *Mochlus sundevallii* (16,984 bp), *Panaspis annettesabinae* (17,176 bp), *Tiliqua occipitalis* (17,085 bp), *Ti*. *scincoides* (17,306 bp), *Trachylepis maculilabris* (17,073 bp), and *Tr*. *striata* (17,130 bp). This combination of newly sequenced and publicly available data maximized the representation of complete mitochondrial sequences across Scincidae subfamilies.

Among the 16 mitogenomes, overall sequence length variation was primarily attributed to differences in the D-loop region. The combined length of the 13 PCGs was highly conserved across species, ranging from 11,319 to 11,406 bp. All mitogenomes contained 13 PCGs, 2 rRNAs, and 22 tRNAs, with identical gene order. *ND6* and eight tRNAs (*trnQ*, *trnA*, *trnN*, *trnC*, *trnY*, *trnS2*, *trnE*, and *trnP*) were located on the light strand, whereas the remaining genes were encoded on the heavy strand, which is consistent with the typical mitogenome architecture observed in Scincidae (Figure S1). The longest intergenic overlaps were consistently observed between *ATP8*-*ATP6* (10 bp) and *ND4L*-*ND4* (7 bp) across most species. The overlap between *ND5* and *ND6* varied from 4 to 16 bp. Major intergenic spacers were present in the regions between *trnN-trnC*, *trnE-CYTB*, *CYTB-trnT*, *ND1*-*trnI*, *trnI-trnQ*, and *trnS2*-*trnD*, exhibiting notable interspecific variation in length (Table S1).

The overall A + T content of the mitogenomes ranged from 55.9% to 61.0%, with AT-skew values between 0.056 and 0.155 and GC-skew values between −0.327 and −0.273. When restricted to the 13 PCGs, A + T content ranged from 55.5% to 60.7%, showing minimal variation compared with the whole mitogenome. AT-skew values for the PCGs ranged from −0.033 to 0.075, indicating a reduced bias toward adenine over thymine, while GC-skew values ranged from −0.338 to −0.277, reflecting a consistent preference for cytosine over guanine (Table S2). Initiation and termination codons usage was analyzed across all PCGs of species. *COX1* consistently used GTG as the initiation codon in all species, whereas the remaining 12 PCGs uniformly initiated with ATG, demonstrating high conservation. Termination codon usage exhibited greater variability. *ATP6*, *ATP8*, and *ND5* consistently terminated with TAA. Other genes used a combination of complete or incomplete stop codons, including TAA, TA, T, AGA, AGG, and TAG. Detailed codon usage patterns are provided in Table S3. Relative Synonymous Codon Usage (RSCU) analysis revealed moderate interspecific variation in codon usage bias, although shared patterns of preferred codon selection were evident (Table S4; Figure S2). The most frequently used codons included CUA (Leu1), CCA (Pro), CGA (Arg), UCA (Ser2), and ACA (Thr), all with RSCU values exceeding 2 in most species. Notably, CGG was not used in *Tr. striata*, where arginine was instead encoded predominantly by CGA and CGC, indicating a species-specific shift in codon preference.

### Deep phylogenetic relationships within Scincidae are sensitive to taxonomic sampling

**Figure 1:**
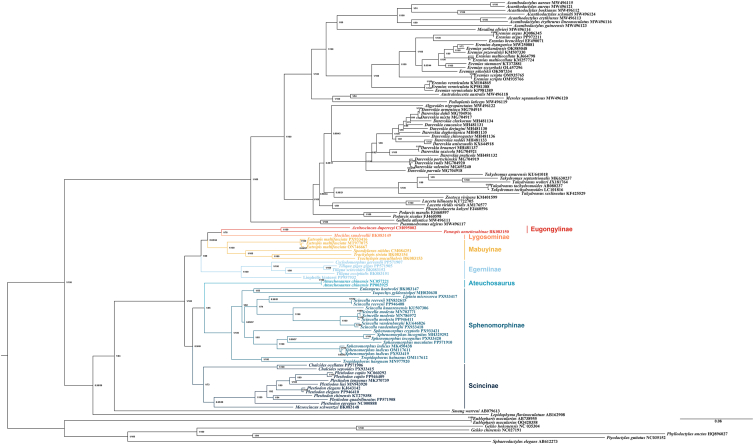
Phylogenetic tree reconstructed from concatenated mitochondrial sequences (PCG12, 12S rRNA, and 16S rRNA) using both BI and ML methods Branch support values indicate PP and BP. Subfamilies within Scincidae are color-coded.

In the initial phylogenetic reconstruction using the full dataset (116 taxa), six subfamilies were recovered within Scincidae, with *Ateuchosaurus* positioned as sister to this clade. Multiple datasets (PCG123, PCG12, PCG123 + rRNA, and PCG12 + rRNA) and analytical conditions (with/without Lacertidae and different outgroups) were explored using both Bayesian inference (BI) and maximum likelihood (ML) methods. However, none of these analyses yielded a consistent and well-supported topology for the deep relationships within the family. Notably, across these analyses, Scincinae and Sphenomorphinae were frequently recovered as sister clades, a result that contradicts the widely accepted phylogeny in which Scincinae occupies the basal position within Scincidae. Given the persistent topological instability under various analytical frameworks, the PCG12 + rRNA dataset was selected for the final presentation of the complete phylogeny, as it provided improved nodal resolution and produced a topology partially congruent with some established relationships (Figure 1). In this topology, ML and BI analyses yielded congruent results for the ingroup. Scincinae and Sphenomorphinae formed a sister clade. Mabuyinae was recovered as the sister group to (Eugongylinae + Lygosominae), albeit with low statistical support (BI posterior probability (PP) = 0.65; ML bootstrap support (BP) = 44), whereas Egerniinae did not form a clade with Mabuyinae. The only notable discrepancy between methods concerned the placement of the outgroup *Eublepharis macularius*: ML placed it within a monophyletic gekkonid clade, whereas BI resolved its position as a polytomy at the base of Gekkota.

**Figure 2:**
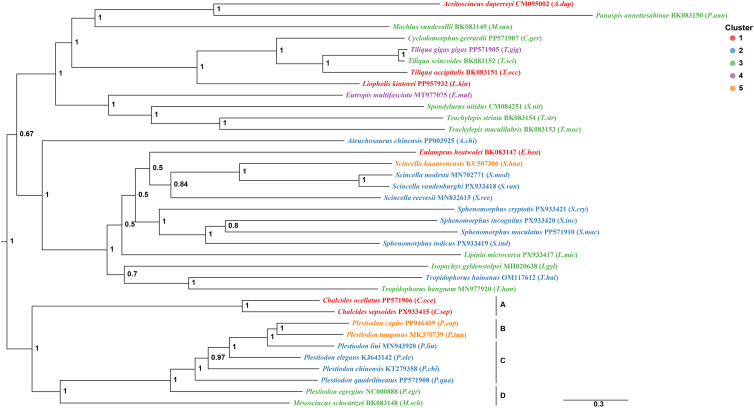
BI tree inferred from 13 PCGs across 38 mitochondrial sequences, with two outgroups deliberately excluded Cluster are color-coded for visual distinction. Within the subfamily Scincinae only, cluster 1 is defined as cluster A, cluster 5 as cluster B, cluster 2 as cluster C, and cluster 3 as cluster D, for the purpose of using these different clusters as foreground branches in selection pressure analysis exclusively within Scincinae.

A separate analysis was subsequently conducted using a reduced, ecologically focused dataset comprising 36 taxa, designed specifically for climatic analyses. The 36 sequences were selected to represent the family Scincidae exclusively and to avoid conspecific sequence duplication. This analysis recovered the classically accepted phylogenetic relationship, with Scincinae placed at the base of Scincidae, Eugongylinae and Lygosominae forming a clade, and Egerniinae resolved as the sister group to this clade (Figure 2). The discrepancies observed between results from the different datasets highlight the sensitivity and instability of deep phylogenetic relationships within Scincidae to taxonomic sampling, underscoring the need for additional genomic and taxonomic data to achieve robust resolution of these nodes.

### Molecular dating based on mitogenomes calibrates the origin times of Scincidae and other major lizard clades

**Figure 3:**
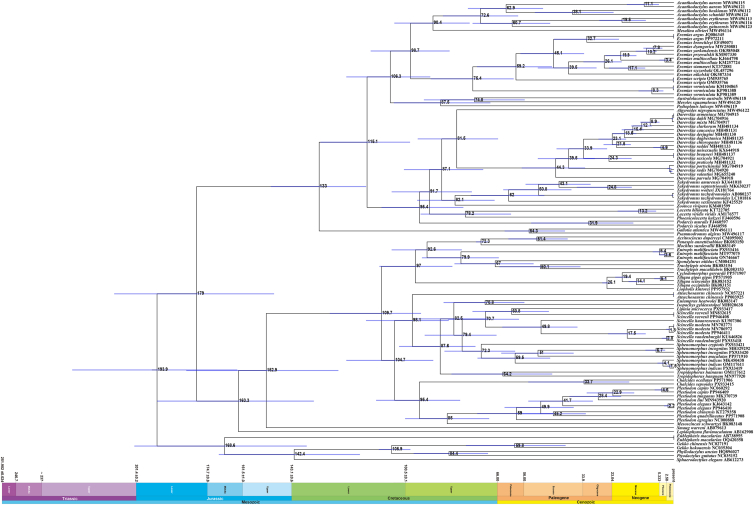
Phylogenetic tree with evolutionary timescale Node values represent median divergence times; blue bars indicate 95% HPD intervals. Geological timescale is calibrated at the base.

Based on divergence time estimation, a subsequent analysis was conducted to examine temporal divergence at the family level. The results indicate that the 95% highest posterior density (HPD) interval for the divergence of Gekkota is estimated at 137.7–202.4 mya. For Scincidae, the 95% HPD for crown-group divergence is dated to 94.9–129.1 mya, and for Lacertidae, to 115.4–151.2 mya. The most recent common ancestor (MRCA) of these three clades is estimated to have existed between 163.7 and 225.7 mya (Figure 3). The addition of Lacertidae species was motivated by the scarcity of fossil evidence within Scincidae. Using fossil calibration points from Lacertidae allowed for more accurate estimation of divergence times for Scincidae and its subfamilies.

### Gene-specific associations between mitochondrial genetic differentiation and climatic variables

**Figure 4:**
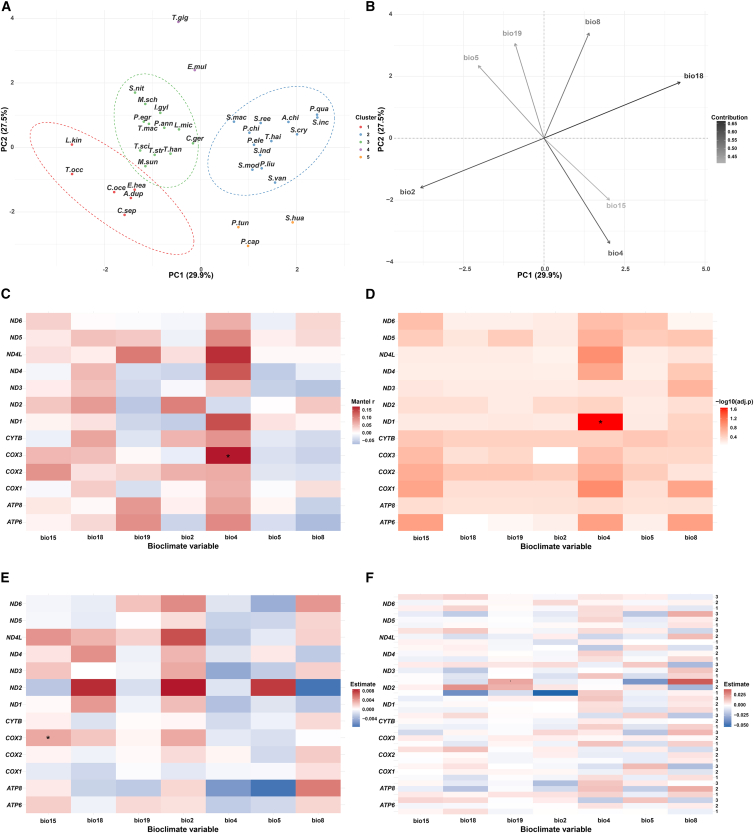
PCA of skink species and correlation analyses between mitochondrial genes and bioclimatic variables (A) PCA based on seven bioclimatic variables (bio2, bio4, bio5, bio8, bio15, bio18, and bio19) for 36 species of Scincidae. The ordination displays projections along the first two principal components, with clusters indicated by color. (B) Variable loading plot corresponding to (A). (C) Mantel tests were used to assess correlations between bioclimatic variables and genetic distances of mitochondrial genes. (D) Distance-based redundancy analysis (dbRDA) was performed to examine correlations between bioclimatic variables and mitochondrial genetic distances while controlling for phylogenetic effects. (E) Associations between bioclimatic variables and average genetic distance were tested using PGLS. (F) PGLS was used to test associations between bioclimate variables and the first three PCoA axes of each gene. The PCoA axis is indicated by the number on the right. Asterisks represent adjusted *p* values less than 0.05.

**Figure 5:**
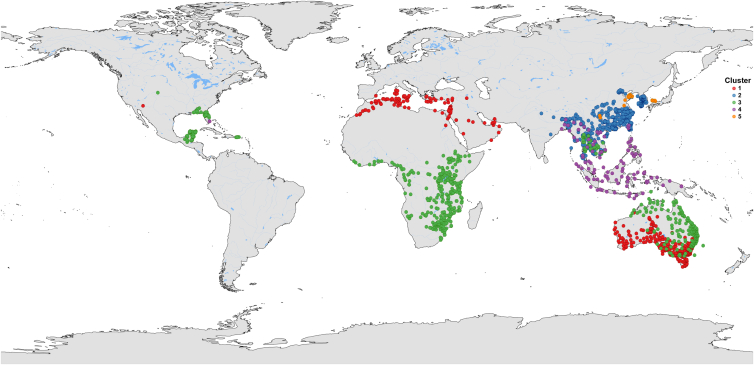
Distribution map of the occurrence records used in this study The species composition within each cluster corresponds to that shown in Figure 4A. See also Table S9.

After removing highly autocorrelated bioclimate variables, seven variables were retained: bio2 (mean diurnal temperature range), bio4 (temperature seasonality), bio5 (maximum temperature of the warmest month), bio8 (mean temperature of the wettest quarter), bio15 (precipitation seasonality), bio18 (precipitation of the warmest quarter), and bio19 (precipitation of the coldest quarter). Principal-component analysis (PCA) of these variables revealed that PC1 and PC2 explained 29.9% and 27.5% of the total variance, respectively. Hierarchical clustering grouped the species into five clusters containing 6, 13, 12, 2, and 3 species, respectively (Figure 4A; Table S5). The different clusters do not form monophyletic groups. Subsequent Mantel tests revealed a significant correlation between bio4 and *COX3* (Mantel *r* = 0.174; adjusted *p* = 0.028; Figure 4C). Distance-based redundancy analysis (dbRDA), which accounts for phylogenetic relationships, identified a significant association between bio4 and *ND1* (*F* = 2.012; adjusted *p* = 0.021; Figure 4D). Phylogenetic generalized least squares (PGLS) analysis detected significant relationships between bio15 with *COX3* (estimate = 0.0035; adjusted *p* = 0.035; Figure 4E) and bio19 with principal coordinate analysis (PCoA) 3 of *ND2* (estimate = 0.0218; adjusted *p* = 0.049; Figure 4F). Based on the results of PCA clustering, the occurrence records of each species were marked on the map with different colors according to their cluster assignments (Figure 5).

### Clade-specific selection analyses identify distinct evolutionary pressures on mitochondrial genes

Based on the grouping results, we performed clade model analyses on the 13 PCGs for each cluster. The results revealed significant purifying selection acting on all genes across all clusters. Notably, when the proportion of certain site classes was zero, it indicated that the estimation of *ω* was unstable, and the proportion of positively selected sites was extremely low (Table 1). In branch-site model analysis, several sites were identified as potentially under positive selection using the Bayes empirical Bayes (BEB) method. Specifically, site 267 threonine (Thr) in *ND1* of cluster 2 was identified as significant. Subsequently, we selected a smaller taxonomic group, Scincinae, to examine positive selection at a finer taxonomic scale. Based on the previous climatic classification, clusters A, B, C, and D were delineated (Figure 2). Branch-site model analyses were conducted separately for each cluster (A, B, C, and D) designated as the foreground branch. In each respective run, the background branches comprised the remaining 36 species in the phylogeny. Positive selection was detected in *ND2* within cluster A, with site 93 proline (Pro) showing a posterior probability >0.95 in the BEB analysis (Table 2). Finally, we performed branch-site unrestricted statistical test for episodic diversification (BUSTED) analysis on all species combined, as well as on each cluster individually as foreground branches. The results indicated that all *p* values were non-significant, suggesting strong purifying selection acting on all branches across each gene (Table 3).

**Table 1:** Clade model analysis of 13 PCGs across climatic groups

Gene	|2ΔlnL|	*p* value^a^	Proportion^b^	*ω*1^c^	*ω*2	*ω*3	*ω*4	*ω*5
*ATP6*	428.346086	0.00000	0.31606	0.06739	0.09078	0.11198	0.14683	0.12272
*ATP8*	217.075758	0.00000	0.45509	0.22519	0.30199	0.22185	0.25118	0.57921
*COX1*	1041.172604	0.00000	0	3.00519	13.86561	12.60001	2.87917	2.23477
*COX2*	283.05931	0.00000	0	3.85267	3.16329	3.42613	1.35691	1.65113
*COX3*	47.730588	0.00000	0.14363	0.09537	0.17364	0.17703	0.11998	0.27621
*CYTB*	493.091758	0.00000	0.18956	0.10305	0.10033	0.14027	0.10364	0.15543
*ND1*	717.713276	0.00000	0	8.83673	20.95496	30.46004	4.96286	10.77612
*ND2*	595.386744	0.00000	0.44383	0.05995	0.10399	0.10589	0.08538	0.12046
*ND3*	539.705412	0.00000	0	1.88953	1.98156	0.35960	0.00010	1.36378
*ND4*	668.251264	0.00000	0	35.56995	48.26688	52.37260	5.81982	2.96933
*ND4L*	193.881574	0.00000	0.37645	0.10866	0.16914	0.11115	0.03517	0.16970
*ND5*	927.373408	0.00000	0	6.35210	17.58442	19.27908	3.10357	3.44714
*ND6*	1267.802998	0.00000	0	4.19371	11.10174	3.05072	2.23738	1.89639

Model comparison: Clade model C (CmC) vs. M2a_rel (number of parameters [np] = 75 vs. 70).

a The *p* value represents the probability obtained from the likelihood ratio test (LRT) comparing the CmC model with the M2a_rel model. A *p* value of zero indicates a statistically significant difference between the models.

b Proportion refers to the estimated fraction of sites subjected to positive selection. In cases where the proportion is zero, the resulting ω can be disregarded, indicating the absence of positively selected sites.

c *ω* denotes the dN/dS ratio.

**Table 2:** Branch-site model analysis of positive selection

Cluster	Gene	Model A LnL	Model A null LnL	|2ΔlnL|	*p*-adjusted^a^	Positive sites (BEB analysis)^a^
1	*ND1*	−15547.08700	−15547.08700	0	1	8 A 0.951∗ 32 I 0.983∗
1	*ND4*	−24781.64371	−24781.64371	0	1	26 M 0.996∗∗ 442 C 0.984∗
1	*ND5*	−33247.28523	−33247.28523	0	1	12 T 0.951∗ 197 M 0.998∗∗ 559 L 0.976∗
2	*ATP6*	−11251.58971	−11251.58971	0	1	168 T 0.997∗∗
2	*ATP8*	−3399.62165	−3399.62165	0	1	47 N 0.967∗
2	*ND1*	−15562.13006	−15556.56692	11.12628	0.02809	267 T 0.967∗
2	*ND2*	−19224.86956	−19224.86956	0	1	218 M 1.000∗∗ 222 T 0.958∗
2	*ND4*	−24777.30292	−24777.30292	0	1	58 I 0.998∗∗ 438 I 0.998∗∗
2	*ND5*	−33238.32639	−33238.32639	0	1	5 P 0.974∗ 47 T 1.000∗∗ 573 N 1.000∗∗
3	*ATP6*	−11254.00401	−11254.00401	0	1	56 L 0.990∗
3	*COX1*	−20314.99341	−20314.99341	0	1	489 S 0.999∗∗ 515 R 0.999∗∗
3	*CYTB*	−17675.64219	−17675.64219	0	1	374 L 0.983∗
3	*ND1*	−15551.61408	−15551.61408	0	1	256 H 0.999∗∗
3	*ND2*	−19213.15612	−19213.15612	0	1	93 P 0.998∗∗ 217 M 1.000∗∗ 313 H 0.998∗∗
3	*ND4*	−24761.95986	−24761.95986	0	1	94 V 0.990∗ 171 M 0.976∗ 179 V 0.980∗ 359 A 0.999∗∗
3	*ND5*	−33228.01266	−33228.01266	0	1	69 N 1.000∗∗ 184 F 1.000∗∗ 186 A 0.963∗ 563 L 0.985∗ 572 T 0.992∗∗
3	*ND6*	−9314.765692	−9314.765692	0	1	101 L 0.992∗∗ 107 G 0.985∗ 109 V 0.999∗∗
5	*COX3*	−10883.21192	−10882.74727	0.92928	1	153 E 0.986∗
5	*CYTB*	−17698.94799	−17698.94799	0	1	244 S 0.990∗
5	*ND1*	−15555.55487	−15557.06952	3.02929	1	320 Q 1.000∗∗
5	*ND2*	−19232.43074	−19232.67424	0.48700	1	4 T 0.979∗ 278 T 0.989∗ 320 K 0.967∗
5	*ND3*	−6475.71110	−6475.71110	0	1	10 T 0.966∗
5	*ND4*	−24787.58404	−24787.58404	0	1	74 V 0.973∗ 191 M 0.974∗
5	*ND4L*	−5383.88615	−5384.04780	0.32329	1	16 M 0.999∗∗
5	*ND5*	−33252.57660	−33252.57660	0	1	409 L 0.963∗ 541 L 0.997∗∗
5	*ND6*	−9331.60076	−9331.61458	0.02765	1	104 L 0.973∗
A	*ND2*	−19233.00533	−19238.71498	11.41929	0.00509	93 P 0.985∗
B	*COX3*	−10885.56891	−10887.26146	3.38509	0.23026	153 E 1.000∗∗
B	*ND6*	−9329.262089	−9330.320451	2.11672	0.33996	104 L 0.996∗∗
C	*COX1*	−20319.17268	−20319.17268	0	1	453 L 0.980∗
C	*ND4*	−24789.85375	−24789.85375	0	1	191 M 0.958∗
D	*CYTB*	−17692.96574	−17692.96574	0	1	107 Y 0.993∗∗
D	*ND2*	−19238.01289	−19238.01464	0.00349	1	336 I 0.981∗

Model comparison: model A vs. model A null (number of parameters [np] = 75 vs. 74). BEB, Bayes empirical Bayes. LnL, Log-likelihood.

a We detected a large number of potentially positively selected sites, with posterior probabilities >0.95 based on BEB analysis. Among these, only the ND1 gene in cluster 2 and the ND2 gene in cluster A had adjusted *p* values <0.05 and were therefore subjected to focused analysis. The *p* value <0.05 justifies the application of the BEB method and supports the existence of positive selection in the dataset, while BEB >0.95 (marked with ∗) and >0.99 (marked with ∗∗) identify the specific residues involved.

**Table 3:** BUSTED analysis for episodic positive selection

Gene	Cluster 1^a^	Cluster 2	Cluster 3	Cluster 4	Cluster 5	All^a^
*ATP6*	1.551 (0%)^b^	3.670 (0.52%)	1.214 (0%)	7.186 (4.41%)	1.664 (0%)	1.787 (0.76%)
*ATP8*	1.146 (10.05%)	2.315 (1.08%)	1.444 (0%)	>100 (1.56%)	1.003 (0%)	2.861 (0%)
*COX1*	1.566 (0.04%)	1.001 (0%)	1.275 (0%)	1.663 (0%)	14.859 (0%)	2.691 (0%)
*COX2*	1.685 (0%)	1.000 (0.14%)	1.729 (0%)	4.613 (1.15%)	1.630 (0%)	2.302 (0%)
*COX3*	1.540 (0%)	>100 (0.23%)	2.804 (0%)	3.529 (3.14%)	>100 (0.21%)	5.659 (0.08%)
*CYTB*	1.860 (0.72%)	1.000 (0.53%)	19.308 (0.28%)	1.432 (4.92%)	1.001 (0%)	2.108 (0.48%)
*ND1*	3.848 (0.36%)	2.497 (0.19%)	1.803 (0.87%)	23.384 (0.41%)	1.001 (0%)	1.213 (0.56%)
*ND2*	1.818 (0%)	1.759 (0.72%)	4.748 (0.65%)	11.030 (0%)	2.550 (0%)	1.787 (1%)
*ND3*	1.584 (0%)	2.183 (0.65%)	1.015 (0%)	54.888 (4.29%)	1.003 (0%)	2.169 (0.11%)
*ND4*	2.228 (1.57%)	1.959 (0%)	1.738 (0.36%)	3.495 (0%)	3.909 (0%)	1.719 (0.32%)
*ND4L*	1.885 (0%)	2.656 (0.56%)	2.331 (0%)	1.417 (0%)	1.988 (0%)	5.69 (0.27%)
*ND5*	2.361 (0.56%)	1.346 (0.47%)	2.408 (1.25%)	1.500 (11.43%)	3.300 (0.59%)	2.083 (0.58%)
*ND6*	7.889 (0.44%)	23.308 (0.16%)	9.471 (1.28%)	2.548 (0%)	1.005 (0%)	6.379 (0.39%)

BUSTED, branch-site unrestricted statistical test for episodic diversification. All *p* values were non-significant after false discovery rate (FDR) correction (adjusted *p* > 0.05).

a Analyses were performed with each cluster and all species combined designated as the foreground branch.

b Values represent the ω estimate under episodic diversifying selection, with the proportion of sites under selection shown in parentheses.

**Figure 6:**
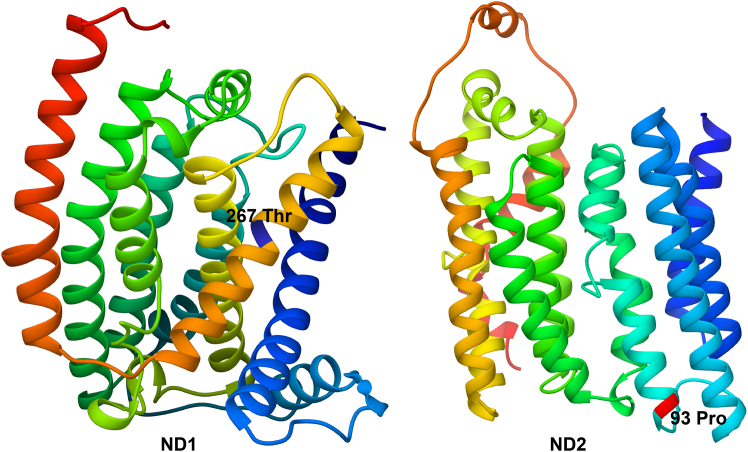
Protein structure diagrams of *ND1* and *ND2* The structure of *ND1* was predicted based on *P. elegans*, and that of *ND2* was predicted based on *C. sepsoides*. The residues 267Thr and 93Pro correspond to the positively selected amino acid sites.

**Figure 7:**
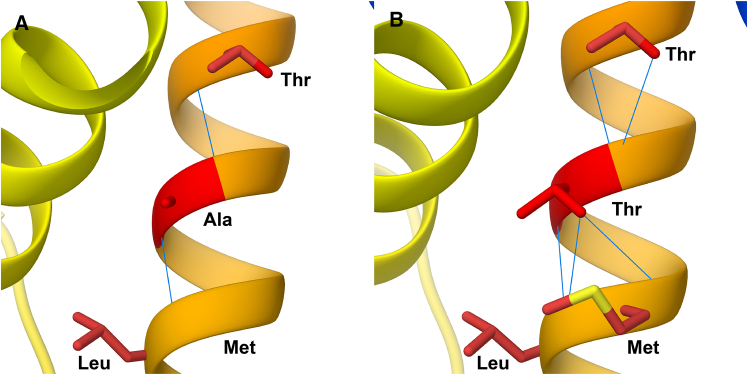
Hydrogen bonding interactions before and after the Thr to Ala substitution in *ND1* (A) Hydrogen bonding interactions of residue 267Ala with surrounding amino acids at site 267 of *ND1* in the foreground branch, predicted using *P. elegans*. Blue lines represent hydrogen bonds. (B) Hydrogen bonding interactions of residue 267Thr with surrounding amino acids at the same site in the background branch, predicted using *T. gigas gigas*. Blue lines represent hydrogen bonds.

The positively selected site in the *ND2* gene is located within a transmembrane helix, corresponding to the initial residues of the fourth helix near the lipid-aqueous interface. At this site, the foreground branches encode glutamic acid (Glu) or glycine (Gly), whereas the background branches encode Pro. In contrast, the positively selected site in the *ND1* gene lies within the central region of a transmembrane helix. Here, the foreground branches possess alanine (Ala), whereas the background branches possess Thr (Figure 6). We subsequently examined the hydrogen bonding interactions formed by the amino acid residues at this site with their surrounding residues, both before and after the amino acid substitution in *ND1* (Figure 7).

## Discussion

Notably, a previous study also recovered Scincinae and Sphenomorphinae as sister clades, although based on limited datasets.^39^ In our initial tree reconstruction, Scincinae and Sphenomorphinae were represented by substantially more species than other subfamilies. This sampling disparity was particularly pronounced for Lygosominae and Eugongylinae, each represented by only one or two species, primarily due to the limited availability of complete mitogenomes for these groups in public databases. We hypothesize that this topology may reflect long-branch attraction (LBA) artifacts,^40^^,^^41^^,^^42^ as when taxon sampling was reduced for Scincinae and Sphenomorphinae in the secondary analysis, these groups no longer formed a clade. Therefore, reconstructing robust phylogenies within Scincidae using complete mitochondrial sequences requires expanded taxonomic sampling with balanced representation across subfamilies. Moreover, the inherently fast evolutionary rate and susceptibility to saturation of mitochondrial datasets may have contributed to these results. Thus, incorporating nuclear gene datasets in future studies could yield more robust phylogenetic resolution.

Subsequent divergence dating estimated the crown age of Squamata at 193.9 mya (163.7–225.7 mya), consistent with recent molecular estimates.^43^^,^^44^ Datta-Roy et al.^45^ recovered Scincidae as a clade originating at 98 mya (84–113 mya) using a dataset of six nuclear loci, including *MKL1*, *PRLR*, *PTGER4*, *R35*, *RAG-1*, and *SNCAIP*. Skinner et al.^46^ estimated its origin at 106 mya (83–131 mya) based on three nuclear genes (*BDNF*, *c-mos*, and *PTPN12*), while Chapple et al.^47^ dated it to 115.8 mya using a mixed dataset combining mitochondrial (*ND2*) and nuclear (*RAG-1*, *c-mos*) genes. In comparison, our mitochondrial-only dataset estimated its origin at 104.7 mya (94.9–129.1 mya). Comparison across these studies suggests that the choice of dataset type (nuclear, mitochondrial, or mixed) did not substantially impact the estimated divergence times. This variation may instead reflect differences in fossil calibrations applied, as well as the taxonomic composition and sampling effort of each study.

By the Mid-Cretaceous, insects had already undergone substantial diversification,^48^ as evidenced by abundant insect fossils preserved in Burmese amber.^49^ This period coincides with the Cretaceous Terrestrial Revolution (KTR, 80–125 mya), during which the radiation of flowering plants triggered major ecological shifts, driving the diversification of pollinators, herbivores, and their predators.^50^ Given that skinks are predominantly insectivorous, the ecological opportunities created by the KTR likely promoted their population expansion and lineage divergence.^51^^,^^52^

In the PCA, we removed climate variables with correlations >0.8 to reduce multicollinearity and used the silhouette coefficient to select the optimal k for hierarchical clustering. This yielded five clusters with uneven species numbers, including two clusters containing only two or three species. To characterize the climatic niches of each cluster, we generated a variable loading plot (Figure 4B) and a distribution map (Figure 5). Species in cluster 1 exhibited higher bio2 values (greater mean diurnal temperature range) and were distributed along the Mediterranean coast, southwestern Australia, and southern Australia. Cluster 2 species were characterized by higher bio18 values, indicating warm-season precipitation consistent with monsoon climate, and were distributed in southeastern and eastern Asia. Cluster 3 species were associated primarily with bio5 (high maximum temperature of the warmest month but low precipitation in the coldest quarter), with distributions largely overlapping tropical savanna regions, mainly in eastern Africa and northern and eastern Australia. Cluster 4 species were characterized by high contributions from both bio5 and bio19, characteristic of tropical rainforest climates, primarily located in the Indonesian Archipelago. Cluster 5 species exhibited high contributions from bio4 and bio15, indicating pronounced temperature and precipitation seasonality, and were also located in the monsoon regions of eastern Asia. These findings suggest that classifying skinks by climate type is informative. However, in Southeast Asia and southeastern Australia, species from different clusters overlapped geographically, indicating limitations in this analytical approach.

To avoid the instability of discrete cluster classification, we treated bioclimatic variables as continuous predictors without species clustering. Subsequently, we performed Mantel tests, dbRDA, and PGLS analyses, with dbRDA and PGLS accounting for phylogenetic relationships among species. All *p* values were corrected using false discovery rate (FDR). The results revealed significant correlations between bio4 and *COX3*, bio15 and *COX3*, and bio4 and *ND1*, but no single variable showed consistent significance across all methods. The *p* value for the association between bio19 and *ND2* PCoA3 in Figure 4F approached 0.05, warranting cautious interpretation. Overall, we did not find widespread correlations between the seven climate variables and mitochondrial genes. The few significant findings were limited to bio4 and bio15. This suggests that temperature and precipitation seasonality may be weakly associated with genetic differentiation in skink mitochondrial genes. Additionally, *COX3* and *ND1* emerge as candidate genes for adaptive evolution under temperature or precipitation variation. We hypothesize that for skinks as ectotherms, temperature seasonality may impose selective pressure on mitochondrial genes involved in energy metabolism. Precipitation seasonality, often linked to primary productivity,^53^ promotes plant growth and consequently increases insect prey availability, which may also exert selective pressure on mitochondrial genes by altering energy demands. In a study on Lacertidae lizards, using the gradient forest approach, the *R*^2^-weighted importance of the *ATP6*, *ATP8*, and *ND3* genes relative to the 19 bioclimatic variables was assessed. Bio3 (isothermality), bio4, bio13 (precipitation of the wettest month), and bio15 emerged as the most influential variables associated with environmental adaptation.^54^ These findings partially align with our results regarding bio4 and bio15, suggesting that these variables may play important roles in driving genetic variation among lizards. However, the contribution of climatic variables should be interpreted with caution, as correlation does not imply causation. In assessments of the combined impacts of deforestation and climate change on the geographic distribution of *Notomabuya frenata* (Mabuyinae, Scincidae), bio3 and bio19 were identified as making the most significant contributions.^55^ This study on South American skinks yielded different results from ours, likely because it did not use mitochondrial gene data but rather used multiple physiological datasets of *N. frenata*. This suggests that the influence of climatic variables on genetic markers differs from their effects on physiological traits and biogeographic distribution patterns, highlighting the importance of dataset type in shaping analytical outcomes.

Clade model and BUSTED analyses revealed that all clusters were under strong purifying selection, indicating significant selective constraints on the skink mitochondrial genome that likely prevent the accumulation of deleterious mutations. In the branch-site model analysis, we detected numerous potentially positively selected sites, two of which reached statistical significance and were examined in detail. Specifically, with species in cluster 2 designated as the foreground branch, a positive selection signal was detected at amino acid site 267 of *ND1*. With species in cluster A (*C. ocellatus* and *C. sepsoides*) served as the foreground branch, a positive selection signal was detected at amino acid site 93 of *ND2*. Species in cluster 2 are primarily distributed in East Asia, mostly within subtropical monsoon climate zones. In contrast, *C. ocellatus* and *C. sepsoides* are mainly distributed in North Africa, the Mediterranean region, and the Arabian Peninsula,^56^^,^^57^ where deserts are widespread.

These results suggest that the hot, arid desert environment and monsoon climate may exert distinct selective pressures on skink lineages inhabiting these regions. The *ND1* and *ND2* gene products are subunits of respiratory complex I, which mediates electron transfer from NADH to ubiquinone and drives proton translocation across the mitochondrial inner membrane.^58^^,^^59^^,^^60^ In *ND2*, the Pro at position 93 was replaced by Gly or Glu. This site is located within the initial residues of an alpha helix. According to the proline rule,^61^^,^^62^ proline at the N1 (N-terminal first) position of an alpha helix enhances protein thermostability due to its rigid pyrrolidine ring, which restricts backbone flexibility.^63^^,^^64^ Proline is indeed frequently observed at the N1 position.^65^ Therefore, while background branches retain Pro at this site, its substitution by Gly/Glu in the desert-adapted lineage suggests that enhanced thermostability was not the primary target of selection. This substitution may lead to increased local flexibility,^66^ potentially enabling the alpha helix to undergo necessary conformational changes more readily under high-temperature conditions, thereby facilitating the survival of species in desert regions. In the *ND1* gene, Thr at position 267 was replaced by Ala, substituting a hydrophilic residue capable of hydrogen bonding (–OH group) with a hydrophobic one. This replacement leads to the loss of hydrogen bonds. Structural analysis revealed that the substitution of Thr with Ala abolishes two hydrogen bonds previously formed with Met at position 264 (Figure 7). This may reduce structural stability while increasing conformational flexibility. Such increased plasticity could facilitate adaptation to humid, seasonally variable monsoon climates, although further experimental validation is required. Indeed, amino acid substitution can lead to partial functional changes in proteins and facilitate adaptation to different environments. For instance, a previous study^67^ identified a single amino acid replacement (Glu183Lys) in the MC1R gene that is differentially fixed between dark and light populations of the lizard *Phrynocephalus erythrurus*, strongly associating with dorsal color variation. Functional assays revealed that this mutation enhances MC1R-α-MSH binding, increases intracellular cyclic AMP (cAMP) levels and cell surface expression, ultimately leading to elevated melanin synthesis and providing a mechanistic link between genotype and adaptive coloration in different environments. In another study,^68^ a conserved amino acid substitution at an allosteric site in *Iguana iguana* hemoglobin is hypothesized to underlie a previously unknown molecular mechanism that confers ADP-specific allosteric regulation. This enables oxygen-binding affinity to respond to metabolic changes (temperature, pH) and thereby facilitating survival across diverse thermal environments.

In summary, in this study, we extracted bioclimatic variables for different skink species and subsequently performed PCA, which grouped these species into five distinct climatic clusters. We then examined the selective pressures acting on skinks within each of these five climatic clusters. Additionally, we conducted correlation analyses between different mitochondrial genes and climatic variables. Furthermore, we reconstructed a phylogenetic tree of skinks based on mitochondrial genes and estimated their divergence times.

### Limitations of the study

Complete mitochondrial genome sequences remain insufficient for the family Scincidae, with particularly sparse representation in several subfamilies. This limited sampling may contribute to the instability observed in the deep-level phylogenetic results. Furthermore, inherent limitations of mitochondrial datasets may also have influenced these phylogenetic inferences, and incorporating nuclear gene datasets in future studies should help resolve these uncertainties. With respect to the correlation analyses, although statistically significant, the correlation values were relatively low. This suggests that the associations between mitochondrial genes and bioclimatic variables, while detectable, likely result from polygenic effects with minor individual contributions. Future studies using more comprehensive approaches are required to substantiate these preliminary correlations. The mitochondrial sequences used were not optimally matched to the occurrence records, which may lead to discrepancies among populations of the same species. Where feasible, population-level analyses would provide more accurate insights into these relationships.

## Resource availability

### Lead contact

Requests for further information and resources should be directed to and will be fulfilled by the lead contact, Danna Yu (ydn@zjnu.cn).

### Materials availability

This study did not generate new unique reagents.

### Data and code availability

• Nucleotide sequence data have been deposited at GenBank: PX933415, PX933416, PX933417, PX933418, PX933419, PX933420, PX933421, PP571910, BK083147, BK083148, BK083149, BK083150, BK083151, BK083152, BK083153, BK083154. The occurrence records dataset is available in Table S9 and has been deposited at Zenodo (https://doi.org/10.5281/zenodo.20839769).
• This paper does not report original code.
• All other data reported in the manuscript will be shared by the lead contact upon request.
• Any additional information required to reanalyze the data reported in this paper is available from the lead contact upon request.

## Acknowledgments

We are grateful to two anonymous reviewers for their comments on a previous version of this manuscript. We thank Huiyuan Wu for his assistance in mitogenome assembly and phylogenetic analysis. This research was supported by the 10.13039/501100001809National Natural Science Foundation of China (no. 31801963) and the 2025 Zhejiang Province College Student Innovation and Entrepreneurship Training Program (S202510345069).

## Author contributions

X.W., L.Z., J.Z., and D.Y. conceived and designed the study; X.W., X.M., and L.Z. developed the methodology; X.W. performed the formal analysis; L.Z., L.D., L.C., R.Z., and J.Z. provided resources; D.Y. validated the findings and administered the project; X.W. wrote the original draft; X.W., J.Z., and D.Y. participated in reviewing and editing the manuscript; X.W. prepared the visualizations; J.Z. supervised the research; D.Y. and X.W. acquired the funding.

## Declaration of interests

The authors declare no competing interests.

## STAR★Methods

### Key resources table

**Table:** 

REAGENT or RESOURCE	SOURCE	IDENTIFIER
**Critical commercial assays**		

Animal Genomic DNA Purification Kit	Sangon Biotech	NO. B518251

**Deposited data**		

*Chalcides sepsoides* mitogenome	this study	GenBank: PX933415
*Eutropis multifasciata* mitogenome	this study	GenBank: PX933416
*Lipinia microcerca* mitogenome	this study	GenBank: PX933417
*Scincella vandenburghi* mitogenome	this study	GenBank: PX933418
*Sphenomorphus indicus* mitogenome	this study	GenBank: PX933419
*Sphenomorphus cryptotis* mitogenome	this study	GenBank: PX933421
*Sphenomorphus incognitus* mitogenome	this study	GenBank: PX933420
*Sphenomorphus maculatus* mitogenome	this study	GenBank: PP571910
*Eulamprus heatwolei* mitogenome	SRR12454791	GenBank: BK083147
*Mesoscincus schwartzei* mitogenome	SRR31187334	GenBank: BK083148
*Mochlus sundevallii* mitogenome	SRR30256543	GenBank: BK083149
*Panaspis annettesabinae* mitogenome	SRR30501129	GenBank: BK083150
*Tiliqua occipitalis* mitogenome	SRR31745583	GenBank: BK083151
*Tiliqua scincoides* mitogenome	SRR31749467	GenBank: BK083152
*Trachylepis maculilabris* mitogenome	SRR30716522	GenBank: BK083153
*Trachylepis striata* mitogenome	SRR30554492	GenBank: BK083154
Occurrence records database (Table S9)	this study	Zenodo: https://doi.org/10.5281/zenodo.20839769

**Software and algorithms**		

fastQC v0.11.6	Andrews et al.^69^	https://www.bioinformatics.babraham.ac.uk/projects/fastqc/
NOVOPlasty v4.2	Dierckxsens et al.^70^	https://github.com/ndierckx/NOVOPlasty
GetOrganelle v1.7.1	Jin et al.^71^	https://github.com/Kinggerm/GetOrganelle
MitoZ v3.6	Meng et al.^72^	https://github.com/linzhi2013/MitoZ
MITOS	Bernt et al.^73^	https://usegalaxy.eu/
PhyloSuite v1.2.3	Xiang et al.^74^	https://github.com/dongzhang0725/PhyloSuite/releases/tag/1.2.3
CGView	Grant et al.^75^	https://proksee.ca/
MAFFT	Katoh et al.^76^	https://mafft.cbrc.jp/alignment/software/
Gblocks	Castresana^77^	https://github.com/atmaivancevic/Gblocks
PartitionFinder2	Lanfear et al.^78^	https://www.robertlanfear.com/partitionfinder/
MrBayes v3.2	Ronquist et al.^79^	http://nbisweden.github.io/MrBayes/
RAxML v8.2	Stamatakis^80^	https://github.com/stamatak/standard-RAxML
PAML v4.10.7	Yang^81^	https://github.com/abacus-gene/paml
Tracer v1.7.2	Rambaut et al.^82^	http://beast.community/tracer
ArcGIS v10.8	Esri	https://www.esri.com
MEGA v11	Tamura et al.^83^	https://www.megasoftware.net/
vegan R package	Oksanen et al.^84^	https://CRAN.R-project.org/package=vegan
ape R package	Paradis et al.^85^	https://CRAN.R-project.org/package=ape
terra R package	Hijmans et al.^86^	https://CRAN.R-project.org/package=terra
caret R package	Max Kuhn^87^	https://CRAN.R-project.org/package=caret
car R package	John et al.^88^	https://CRAN.R-project.org/package=car
cluster R package	Maechler et al.^89^	https://CRAN.R-project.org/package=cluster
phylolm R package	Ho et al.^90^	https://CRAN.R-project.org/package=phylolm
HyPhy v2.5	Kosakovsky Pond et al.^91^	https://github.com/veg/hyphy
SWISS-MODEL	Waterhouse et al.^92^	https://swissmodel.expasy.org/
UCSF Chimera v1.19	Pettersen et al.^93^	https://www.cgl.ucsf.edu/chimera/

**Other**		

Illumina sequencing	Beijing BerryGenomics Co., Ltd.	Illumina HiSeq 2000 platform

### Experimental model and study participant details

All animal care protocols were approved by the Animal Care Committee of Zhejiang Normal University (protocol # ZSDW2024038). Skinks were collected, transported to the laboratory, and housed in identical plastic incubators (120 × 90 × 110 cm) under controlled conditions (25°C, 12 h light/dark cycle). They were fed a diet of mealworms (*Tenebrio molitor*). Water was regularly provided in a Petri dish, and occasionally the inner walls of the enclosures were sprayed with water to maintain humidity. A small piece of tail tissue was clipped from each skink for mitochondrial DNA analysis. Because mitochondrial genome sequences are generally considered to be stably inherited and not known to vary systematically with sex or age, a formal statistical analysis for the influence of sex or developmental stage on the sequence data was not performed.

### Method details

#### Sample collection and DNA extraction

Specimens were collected from the following localities: *C*. *sepsoides* (Abu Rawash, Giza, Egypt), *Eut*. *multifasciata* (18°54′*N* 109°41′E, Wuzhishan, Hainan, China), *L*. *microcerca* (Phuc-son, Annam, Vietnam), *Sc*. *vandenburghi* (32°04′*N* 118°32′E, Nanjing, Jiangsu, China), *Sp*. *indicus* (29°57′*N* 120°33′E Shaoxing, Zhejiang, China), *Sp*. *cryptotis* (21°30′E 107°56′N, Shiwandasha, Guangxi, China), *Sp*. *incognitus* (23°10′*N* 113°17′E, Guangzhou, Guangdong, China), and *Sp*. *maculatus* (23°55′*N* 106°35′E, Baise, Guangxi, China). The specimens of the non-native species *C*. *sepsoides* and *L*. *microcerca* were collected in 2010 and are currently deposited in the Zoological Museum of Zhejiang Normal University under voucher numbers ZJNU-20100712-DWSX 012 and ZJNU-20100712-DWSX 013, respectively. The remaining six species were collected during the fieldwork from 2022 to 2024. All fluid-preserved specimens and freshly captured specimens were photographed prior to tissue sampling. Genomic DNA was then extracted from tail-clip samples using the Ezup Column Animal Genomic DNA Purification Kit (Sangon Biotech, Shanghai, China) according to the manufacturer’s instructions.

#### Mitogenome sequencing, assembly, and annotation

DNA samples with concentrations exceeding 25 μg/mL were submitted to Berry Genomics (Beijing, China) for next-generation sequencing. High-throughput sequencing was performed on the Illumina HiSeq 2000 platform (Illumina, Inc., San Diego, CA, USA) using a 200 bp paired-end sequencing strategy. The quality of raw sequencing data was initially assessed using FastQC v0.11.6. Subsequently, low-quality reads and adapter sequences were filtered out using fastp. Sequencing data for additional species were retrieved from the SRA database: *Eul*. *heatwolei* (SRR12454791),^94^ *Me*. *schwartzei* (SRR31187334),^95^ *Mo*. *sundevallii* (SRR30256543),^95^ *P*. *annettesabinae* (SRR30501129),^95^ *Ti*. *occipitalis* (SRR31745583), *Ti*. *scincoides* (SRR31749467), *Tr*. *maculilabris* (SRR30716522),^95^ and *Tr*. *striata* (SRR30554492).^95^ A total of 16 mitochondrial genomes, including those newly generated and previously published sequences, were assembled using NOVOPlasty v4.2,^70^ GetOrganelle v1.7.1,^71^ or MitoZ v3.6^72^ to ensure data accuracy and integrity. For all assemblies, the default parameters of each software package were used. The gene coordinates of all 37 mitochondrial genes were annotated using MITOS WebServer (https://usegalaxy.eu/, accessed on 26 June 2025)^73^ followed by manual curation to ensure annotation accuracy. Genomic features such as intergenic overlaps, intergenic spacers, A + T content, AT-skew, GC-skew, and RSCU values were calculated using PhyloSuite v1.2.3.^74^ Complete mitogenome maps were visualized using the CGView online server (https://proksee.ca/, accessed on 26 June 2025).^75^

#### Phylogenetic tree construction

A total of 100 additional mitochondrial sequences were downloaded from the NCBI database and combined with the 16 previously obtained sequences, resulting in a final total of 116. The final dataset included 59 Lacertidae sequences,^96^^,^^97^^,^^98^^,^^99^^,^^100^^,^^101^^,^^102^^,^^103^^,^^104^^,^^105^^,^^106^^,^^107^^,^^108^^,^^109^^,^^110^^,^^111^^,^^112^^,^^113^^,^^114^^,^^115^^,^^116^^,^^117^^,^^118^ 50 Scincomorpha sequences,^34^^,^^119^^,^^120^^,^^121^^,^^122^^,^^123^^,^^124^^,^^125^^,^^126^^,^^127^^,^^128^^,^^129^^,^^130^^,^^131^^,^^132^^,^^133^^,^^134^^,^^135^^,^^136^^,^^137^ and seven Gekkota sequences^138^^,^^139^^,^^140^^,^^141^ (Table S6). The 13 PCGs and two rRNAs were extracted from all sequences, individually aligned using MAFFT,^76^ trimmed with Gblocks^77^ to remove ambiguously aligned regions, and concatenated into a single supermatrix using PhyloSuite v1.2.3. PartitionFinder2^78^^,^^142^ was applied to determine the optimal partitioning schemes (the first and second codon positions were used for one analysis of 116 taxa, while the complete coding sequence was used for the second analysis of 38 taxa) and best-fit nucleotide substitution models (Table S7). Bayesian Inference (BI) analysis was performed in MrBayes v3.2^79^ under the selected models, with 10 million generations with trees sampled every 1,000 generations. The initial 25% of sampled generations were discarded as burn-in, and convergence was confirmed by ensuring that the average standard deviation of split frequencies remained below 0.01. Maximum Likelihood (ML) analysis was conducted in RAxML v8.2^80^ with 1,000 bootstrap replicates to evaluate nodal support.

In the initial phylogenetic analysis, ML and BI trees were constructed using a dataset comprising first- and second-codon positions of the 13 PCGs (PCG12) and rRNA sequences from 116 taxa. For the subsequent analysis, the dataset was restricted to species within Scincidae, and putative paralogous sequences were excluded, resulting in a refined set of 36 ingroup taxa. A new BI analysis was then conducted using the 13 mitochondrial PCGs from these 36 species along with two outgroup species (*Smaug warreni*^114^ and *Lepidophyma flavimaculatum*^123^) to reconstruct the phylogenetic relationships within the family Scincidae.

#### Divergence time estimation

Divergence times were estimated using the mcmctree module in PAML v4.10.7,^81^ with Markov chain Monte Carlo sampling conducted over 1 million generations and the initial 40% of samples discarded as burn-in. Four fossil-based calibration points were applied to constrain node ages. To compensate for the limited availability of reliable calibration points within Scincidae, external calibrations from Lacertidae and Gekkota were also incorporated. Following Heinicke et al.,^143^ the divergence between Gekkonidae and Phyllodactylidae was calibrated to a uniform prior of 97–110 Mya using an amber-preserved fossil representing the crown group Gekkota.^144^ Following Salvi et al.,^145^ the split between Gallotiinae and Lacertinae was constrained to a prior range of 40.4–150 Mya using the fossil *Plesiolacerta lydekkeri* (assigned to Lacertinae)^146^ and molecular estimates from the median crown age of Lacertoidea.^147^ The crown age of the Gallotiinae clade was calibrated to 28.1–61.6 Mya using both minimum and maximum fossil constraints.^148^^,^^149^ Following Ghosh et al.,^150^ the most recent common ancestor (MRCA) of Egerniinae was constrained to a uniform prior of 12.5–25 Mya. We used uniform distributions for fossil calibrations and employed the Birth-Death Process as the tree prior. All resulting time estimates were evaluated in Tracer v1.7.2^82^ to confirm convergence and adequate sampling, with effective sample size (ESS) values exceeding 200 for all parameters (Table S8).

#### Climate analyses

Bioclimatic variables are derived from monthly total precipitation, monthly minimum temperature, and monthly maximum temperature, resulting in 19 variables that provide a basis for identifying key factors influencing species’ geographic distribution and environmental adaptation.^151^ We downloaded 19 bioclimatic layers from WorldClim 2.1^151^ (https://worldclim.org/data/, accessed on 20 June 2025) at a spatial resolution of 2.5 arc-minutes. Occurrence records for 36 species of Scincidae were subsequently obtained from GBIF^152^ (https://www.gbif.org/, accessed on 4 March 2026). To mitigate sampling bias, the occurrence data were thinned using a grid-based approach. A total of 2,341 occurrence points were retained after processing (Table S9). Species with fewer than five occurrence points included *I*. *gyldenstolpei*, *Pa*. *annettesabinae*, *Pl*. *tunganus*, and *Tro*. *hangnam*. For each species, the maximum number of occurrence points retained was limited to 100. A distribution map of occurrence records was generated using ArcGIS v10.8 (Esri, Redlands, CA, USA). The 19 bioclimatic variables were extracted for each occurrence point using the terra^86^ package in R. For each species, the mean value of each bioclimatic variable was calculated across all its occurrence points to represent the species’ climatic profile. To reduce multicollinearity among the 19 bioclimatic variables, a correlation analysis was performed using the caret^87^ R package. Variables with pairwise correlation coefficients greater than 0.8 were removed, retaining the following seven variables: bio2, bio4, bio5, bio8, bio15, bio18, and bio19. Variance Inflation Factor (VIF) for the remaining variables was calculated using the car^88^ package (with bio15 as the reference variable) to assess multicollinearity. All VIF values were below 5, except for bio18, which was slightly below 10 (Table S10). A PCA was subsequently conducted based on these seven bioclimatic variables, with data standardization applied prior to analysis. Hierarchical clustering was performed using the cluster^89^ R package, with the optimal number of clusters (k) automatically selected based on silhouette coefficients. Following cluster determination, 95% confidence ellipses and corresponding variable loading plots were generated for each cluster.

Each mitochondrial gene was individually aligned using MAFFT as implemented in MEGA v11.^83^ Genetic distance matrices for each gene were subsequently generated by calculating p-distances. Mantel tests, implemented in the vegan^84^ R package, were employed to assess pairwise correlations between each bioclimatic variable and the genetic distance matrices. The resulting *p*-values were adjusted using the FDR. Subsequently, a dbRDA was conducted using phylogenetic principal coordinates calculated from the phylogenetic tree using the ape^85^ package to account for phylogenetic non-independence. The dbRDA was used to analyze correlations between climatic variables and genetic distances, incorporating the first five phylogenetic principal coordinates as conditional variables to control for phylogenetic effects. Finally, *p*-values were subsequently adjusted using the FDR. We performed PCoA on each gene’s genetic distance matrix to extract the first three axes and calculated average genetic distance per species. PGLS models with Brownian motion (BM) evolution were then fitted using the phylolm^90^ R package, regressing these genetic diversity metrics against climate variables, with *p*-values adjusted using FDR. We performed PGLS analyses using the BM, OUfixedRoot, and lambda models, respectively. The estimate of λ in the lambda model hit the boundary of the parameter space, so the model was discarded. This behavior suggests that the BM model is likely more suitable. The BM model produced the lowest Akaike Information Criterion (AIC) value and was therefore selected.

#### Selection pressure analyses

Selection pressure analysis was conducted using CodeML in PAML v4.10.7 based on the BI tree after removing outgroup taxa. Five foreground branches, corresponding to the climatic groups, were designated on this topology. For each PCG, a Clade Model C (CMC) analysis was performed to compare the alternative model (CMC) against the restricted null model (M2a_rel). Branch-site Model A analysis was then performed individually for each climatic group, comparing the alternative model (model A) with its null counterpart (model A null) to detect episodic positive selection. Selective pressure across groups was quantified using the *ω* ratio (*dN*/*dS*), where *ω* = 1 indicates neutral evolution, *ω* > 1 suggests positive selection, and *ω* < 1 denotes purifying selection. The statistical significance of differences between alternative and null models was assessed using a likelihood ratio test (LRT), with *p*-values <0.05 considered statistically significant after FDR correction where applicable. Subsequently, selection pressure analysis was performed using BUSTED implemented in HyPhy v2.5^91^ for all branches of each gene within each cluster, as well as across all species collectively. *p*-values were adjusted using FDR. 3D protein structure prediction was performed using SWISS-MODEL^92^ (https://swissmodel.expasy.org/, accessed on 20 July 2025) based on available homologous templates. We used UCSF Chimera v1.19^93^ to visualize protein structures and identify potential hydrogen bonds.

### Quantification and statistical analysis

All statistical analyses were performed in R v4.4.3, and the FDR method was used for *p*-value adjustment. A *p* value <0.05 was considered statistically significant. Exact *p* values are reported in the tables or results unless *p* < 0.00001. All statistical tests used are indicated in the respective figure legends or tables (denoted as a, b, c). For phylogenetic reconstruction, datasets comprising 116 sequences and 38 sequences were used. For selection pressure analyses, 36 sequences from different species were used to test each of the 13 mitochondrial genes, with the species of interest designated as the foreground branch and all remaining species serving as the background branch.

## Supplemental information

Document S1. Figures S1 and S2 and Tables S1–S5, S7, and S10

Table S6. The species names and accession names included in this phylogenetic tree, related to STAR Methods

Table S8. Effective sample size (ESS) values estimated in the divergence time analysis, related to Figure 3

Table S9. Occurrence records for 36 skink species and retained after thinning, related to Figure 5
