# Supplementary material for: Climate-driven selection may shape mitochondrial genome evolution in Scincidae
Source: iScience. 2026 Jul 14;29(8):116789. doi: 10.1016/j.isci.2026.116789 (PMC13382602; doi:10.1016/j.isci.2026.116789)
Supplement: Document S1. Figures S1 and S2 and Tables S1–S5, S7, and S10 [file mmc1.pdf]

iScience, Volume *29*

Supplemental information

Climate-driven selection may shape mitochondrial genome evolution in Scincidae

Xuxiang Wu, Lemei Zhan, Lingyi Ding, Lining Chen, Runan Zheng, Xingzhou Ma, Jiayong Zhang, and Danna Yu

Supplementary Figures

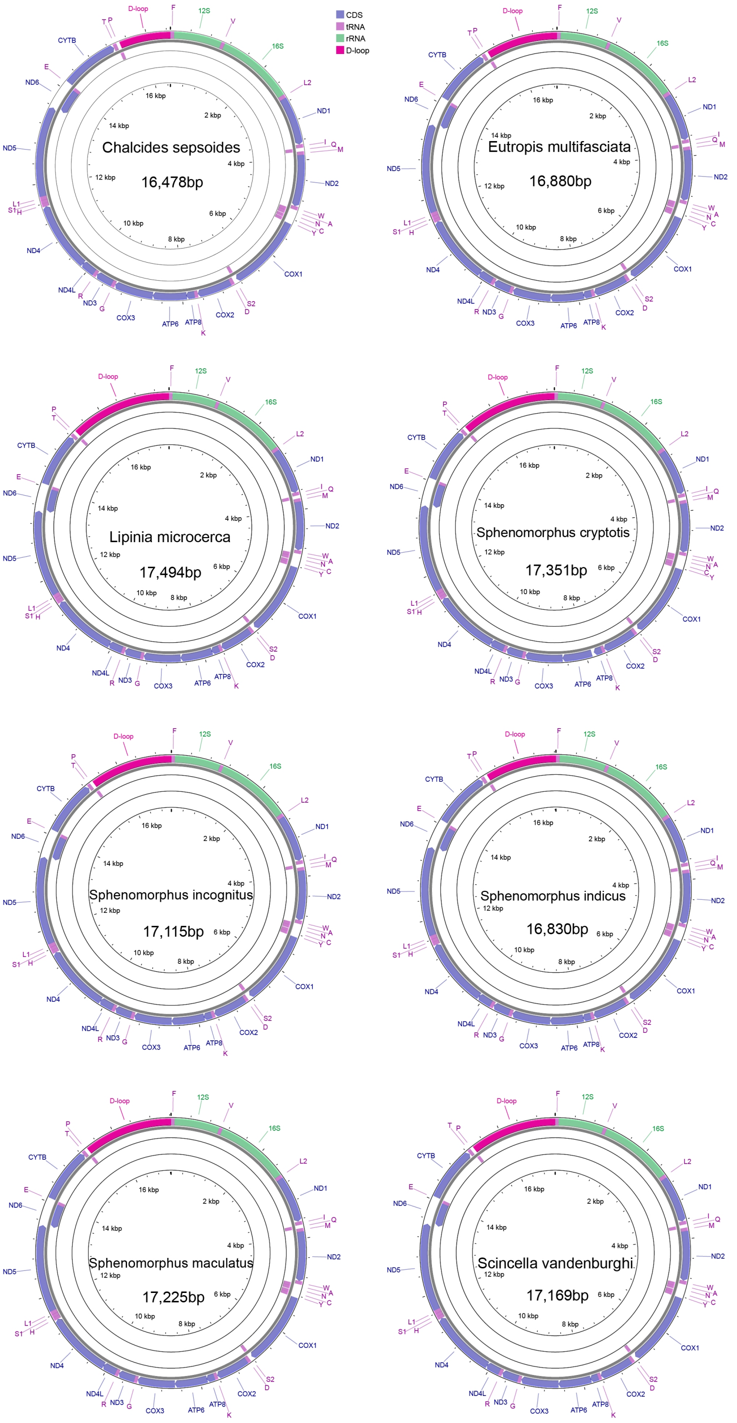

Figure S1. Depicted are the circular mitogenome maps for eight species characterized in this investigation. The two peripheral rings illustrate the genomic architecture, outer ring for heavy-strand genes, inner ring for light-strand genes, with tRNAs labeled via conventional abbreviations.

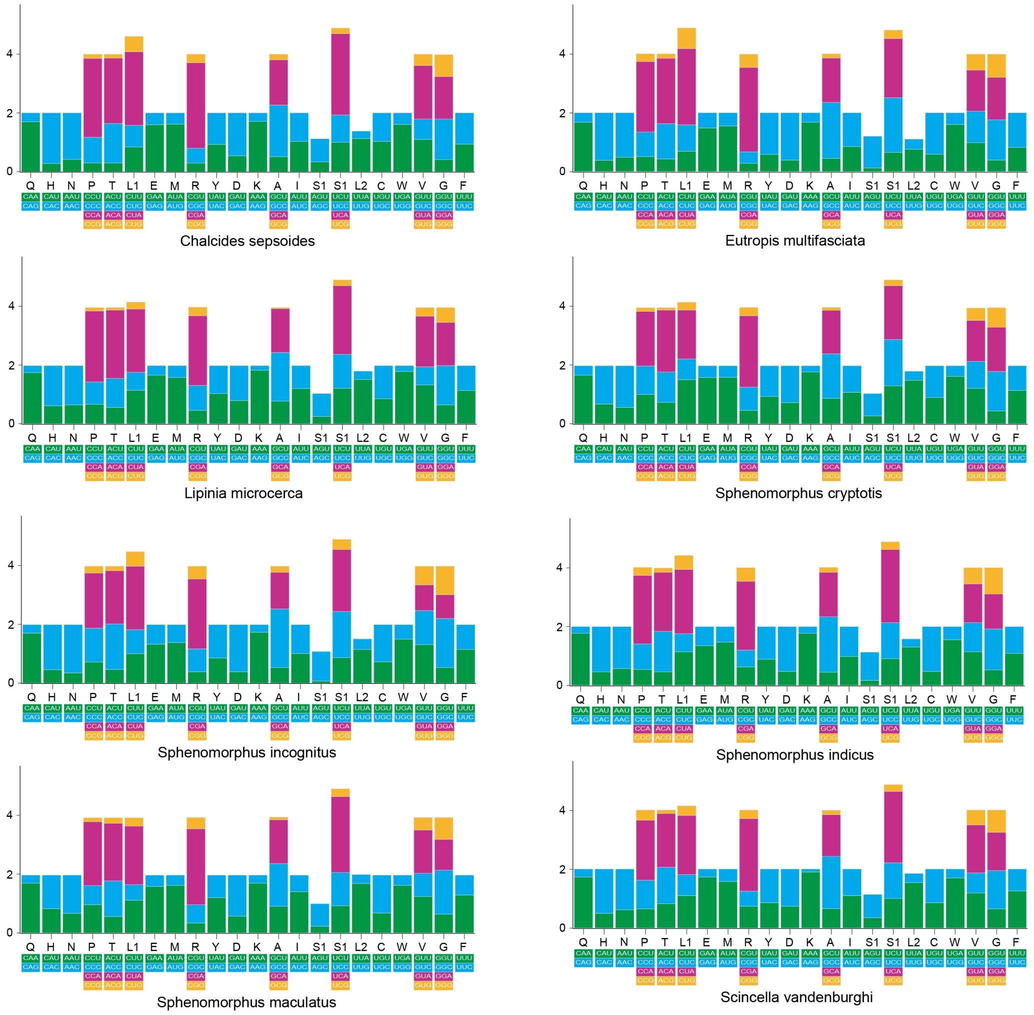

Figure S2. Relative synonymous codon usage (RSCU) for the eight mitogenomes. The abscissa lists all synonymous codon families, with each triplet represented by a separate color. The ordinate gives the corresponding RSCU values. Codon identity is indicated by the color key.

Supplementary Tables

Table S1. Lengths of intergenic overlaps and spacers in the mitogenomes of the eight sequenced skink species, Related to Figure S1.

Genes	*C. sepsoides*	*E. multifasciata*	*L. microcerca*	*S. vandenburghi*	*S. indicus*	*S. cryptotis*	*S. incognitus*	*S. maculatus*	*A. duperreyi*	*E. heatwolei*	*M. schwartzei*	*M. sundevallii*	*P. annettesabinae*	*T. occipitalis*	*T. scincoides*	*T. maculilabris*	*T. striata*
F	0[a]	0	1	1	0	0	0	0	0	0	0	0	0	0	0	0	0
12S	0	-3	0	0	-1	-3	-2	0	1	-1	0	-3	-2	-1	-1	-1	-1
V	0	-1	0	0	1	2	2	0	2	2	2	1	2	2	2	-3	-6
16S	1	0	0	0	0	0	0	0	4	1	1	3	3	2	3	2	2
L2	0	0	0	0	0	0	0	0	1	0	0	1	1	1	1	1	1
ND1	2	2	-2	2	4	4	2	6	9	4	4	7	7	4	4	2	-1
I	3	6	0	1	2	-2	0	3	1	2	5	1	1	9	13	2	1
Q	-1	-1	-1	-1	-1	-1	-1	-1	-1	-1	-1	-1	-1	-1	-1	-1	-1
M	0	0	0	0	0	0	0	0	0	0	0	0	0	0	0	0	0
ND2	0	0	1	0	-2	0	-1	-2	0	-1	1	0	-2	-2	-1	2	-2
W	-1	0	-1	-1	0	-1	-1	-1	-1	0	-1	-1	-1	-1	-1	-1	-1
A	0	0	0	0	0	0	0	0	0	0	0	0	0	0	0	0	0
N	11	19	17	16	13	15	13	14	7	14	14	16	1	18	18	23	17
C	8	0	0	0	0	0	0	0	0	0	0	1	0	3	4	15	0
Y	1	1	1	1	1	1	1	1	142	1	1	1	135	1	1	1	1
COX1	-5	-5	-5	-5	-5	-5	-5	-5	-9	-5	-5	-5	-5	-5	-5	-5	-9
S2	10	8	3	3	3	3	4	3	3	3	3	3	3	2	2	3	3
D	1	1	1	1	0	1	0	0	1	1	1	1	1	2	2	1	1
COX2	0	0	0	0	0	0	0	0	0	0	0	0	0	0	0	0	-2
K	1	1	0	0	2	1	2	2	1	1	1	1	1	1	1	1	1
ATP8	-10	-10	-10	-10	-10	50	-10	-10	-10	-10	-10	-10	-10	-10	-10	-10	-10

ATP6	-1	-1	-1	-1	-1	-1	-1	-1	-1	-1	-1	-1	-1	-1	-1	-1	-1
COX3	0	0	0	0	0	0	0	0	-1	0	0	0	-1	-1	0	-1	-1
G	0	0	1	0	0	0	0	0	0	0	0	0	0	0	0	0	0
ND3	-1	0	0	0	-2	0	-2	-2	0	-2	-2	0	0	-2	-2	-2	-2
R	2	0	0	0	0	0	0	0	0	0	0	0	0	0	0	0	0
ND4L	-7	-7	-7	-7	-7	-7	-7	-7	-7	-7	-7	-7	-7	-7	-7	-7	-7
ND4	0	0	0	0	0	0	0	0	0	0	0	0	0	0	0	0	0
H	0	0	0	0	0	0	0	0	0	0	0	0	0	0	0	0	0
S1	-1	-1	-1	-1	-1	-1	-1	-1	-1	-1	-1	-1	-1	-1	-1	-1	-1
L1	1	1	1	1	1	1	1	1	1	1	1	1	1	1	1	1	1
ND5	-4	-4	-5	-5	-5	-5	-5	-5	-4	-5	-4	-5	-4	-5	-5	-5	-16
ND6	0	0	0	0	0	-3	0	0	0	0	0	1	0	0	0	0	0
E	3	2	2	2	2	3	2	2	2	1	2	2	2	2	2	2	3
CYTB	4	7	3	9	6	7	6	-1	3	7	7	6	3	9	11	12	10
T	0	0	0	0	0	0	0	0	0	0	0	0	0	0	0	0	0
P	0	0	0	0	0	0	0	0	0	0	0	0	0	0	0	10	0
D-loop																	

[a]The values represent the number of bases, positive values indicate spacers between genes, and negative values indicate overlaps.

Table S2. Sequence characteristics of mitochondrial genomes and PCGs for 17 species: Length, AT content[a], AT-skew[b], and GC-skew[c].

Species	Whole Genome				PCGs			
	Length (bp)	A+T%	AT-K	GC-K	Length (bp)	A+T%	AT-K	GC-K
Chalcides sepsoides	16,478	57.9	0.155	-0.326	11,391	57.8	0.073	-0.325
Eutropis multifasciata	16,880	56.4	0.140	-0.316	11,385	55.8	0.075	-0.317
Lipinia microcerca	17,494	60.2	0.112	-0.323	11,382	60.4	0.029	-0.313
Scincella vandenburghi	17,169	59.0	0.085	-0.300	11,379	58.9	0.005	-0.308
Sphenomorphus indicus	16,830	58.0	0.114	-0.309	11,382	57.3	0.031	-0.310
Sphenomorphus cryptotis	17,351	59.3	0.073	-0.316	11,319	59.0	-0.018	-0.326
Sphenomorphus incognitus	17,115	56.2	0.095	-0.314	11,385	55.5	0.002	-0.307
Sphenomorphus maculatus	17,225	61.0	0.075	-0.273	11,385	60.6	-0.007	-0.277
Eulamprus heatwolei	17,116	60.1	0.096	-0.281	11,385	59.7	0.011	-0.281
Mesoscincus schwartzei	16,897	57.1	0.142	-0.327	11,388	57.3	0.060	-0.336
Mochlus sundevallii	16,984	55.9	0.147	-0.327	11,385	55.9	0.068	-0.338
Panaspis annettesabinae	17,176	58.7	0.056	-0.322	11,382	58.1	-0.033	-0.325
Tiliqua occipitalis	17,085	55.9	0.096	-0.323	11,373	55.7	0.008	-0.337
Tiliqua scincoides	17,306	57.2	0.073	-0.311	11,376	57.5	-0.004	-0.331
Trachylepis maculilabris	17,073	58.1	0.081	-0.307	11,388	58.2	-0.013	-0.316
Trachylepis striata	17,130	60.2	0.084	-0.313	11,406	60.7	-0.006	-0.311

[a]AT content = (A+T)/(A+T+G+C) × 100%

[b]AT-skew = (A−T)/(A+T)

[c]GC-skew = (G−C)/(G+C)

Table S3. Usage patterns of start and stop codons in 13 PCGs across 16 mitochondrial sequences.

Genes		*C. sepsoides*	*E. multifasciata*	*L. microcerca*	*S. vandenburghi*	*S. indicus*	*S. cryptotis*	*S. incognitus*	*S. maculatus*	*E. heatwolei*	*M. schwartzei*	*M. sundevallii*	*P. annettesabinae*	*T. occipitalis*	*T. scincoides*	*T. maculilabris*	*T. striata*
start codon	*ATP6*	ATG	ATG	ATG	ATG	ATG	ATG	ATG	ATG	ATG	ATG	ATG	ATG	ATG	ATG	ATG	ATG
	ATP8	ATG	ATG	ATG	ATG	ATG	ATG	ATG	ATG	ATG	ATG	ATG	ATG	ATG	ATG	ATG	ATG
	COX1	GTG	GTG	GTG	GTG	GTG	GTG	GTG	GTG	GTG	GTG	GTG	GTG	GTG	GTG	GTG	GTG
	COX2	ATG	ATG	ATG	ATG	ATG	ATG	ATG	ATG	ATG	ATG	ATG	ATG	ATG	ATG	ATG	ATG
	COX3	ATG	ATG	ATG	ATG	ATG	ATG	ATG	ATG	ATG	ATG	ATG	ATG	ATG	ATG	ATG	ATG
	ND1	ATG	ATG	ATG	ATG	ATG	ATG	ATG	ATG	ATG	ATG	ATG	ATG	ATG	ATG	ATG	ATG
	ND2	ATG	ATG	ATG	ATG	ATG	ATG	ATG	ATG	ATG	ATG	ATG	ATG	ATG	ATG	ATG	ATG
	ND3	ATG	ATG	ATG	ATG	ATG	ATG	ATG	ATG	ATG	ATG	ATG	ATG	ATG	ATG	ATG	ATG
	ND4	ATG	ATG	ATG	ATG	ATG	ATG	ATG	ATG	ATG	ATG	ATG	ATG	ATG	ATG	ATG	ATG
	ND4L	ATG	ATG	ATG	ATG	ATG	ATG	ATG	ATG	ATG	ATG	ATG	ATG	ATG	ATG	ATG	ATG
	ND5	ATG	ATG	ATG	ATG	ATG	ATG	ATG	ATG	ATG	ATG	ATG	ATG	ATG	ATG	ATG	ATG
	ND6	ATG	ATG	ATG	ATG	ATG	ATG	ATG	ATG	ATG	ATG	ATG	ATG	ATG	ATG	ATG	ATG
	CYTB	ATG	ATG	ATG	ATG	ATG	ATG	ATG	ATG	ATG	ATG	ATG	ATG	ATG	ATG	ATG	ATG
terminal codon	*ATP6*	TAA	TAA	TAA	TAA	TAA	TAA	TAA	TAA	TAA	TAA	TAA	TAA	TAA	TAA	TAA	TAA
	ATP8	TAA	TAA	TAA	TAA	TAA	TAA	TAA	TAA	TAA	TAA	TAA	TAA	TAA	TAA	TAA	TAA
	COX1	AGA	AGA	AGA	AGA	AGA	AGA	AGA	AGA	AGA	AGA	AGA	AGA	AGA	AGA	AGA	AGG
	COX2	T	T	T	T	T	T	T	T	T	T	T	T	T	T	T	TAA
	COX3	T	T	T	T	T	T	T	T	T	T	T	TA	TA	T	TA	TA
	ND1	TAA	TAA	TAG	TAA	TAA	TAA	TAA	TAA	TAA	TAG	TAA	TAA	TAA	TAA	T	T
	ND2	T	T	T	T	TAG	T	TAA	TAG	TAA	TAG	TAA	TAA	TAA	TAA	TAA	TAG

ND3	TAA	T	T	T	TAA	T	TAA	TAA	TAA	TAA	TAA	TAG	TAG	TAA	TAA	TAA
ND4	T	T	T	T	T	T	T	T	TAA	TAA	T	T	TAG	TAG	TAA	TAA
ND4L	TAA	TAA	TAA	TAA	TAA	TAA	TAA	TAA	T	T	T	T	T	T	T	T
ND5	TAA	TAA	TAA	TAA	TAA	TAA	TAA	TAA	TAA	TAA	TAA	TAA	TAA	TAA	TAA	TAA
ND6	TAG	TAG	AGG	AGG	AGG	AGG	AGG	AGG	TAA	TAA	TAA	TAA	TAA	TAA	TAA	AGA
CYT B	TAG	TAA	TAA	TAA	TAA	TAA	TAA	TAG	AGG	TAA	AGA	TAG	AGG	AGA	AGG	AGA

Table S4. RSCU values[a] for all codons across 16 mitogenomic sequences, Related to Figure S2.

AA	Codon	*E. multifasciata*	*C. sepsoides*	*L. microcerca*	*S. vandenburghi*	*S. indicus*	*S. cryptotis*	*S. incognitus*	*S. maculatus*	*A. duperreyi*	*E. heatwolei*	*M. schwartzei*	*M. sundevallii*	*P. annettesabinae*	*T. occipitalis*	*T. scincoides*	*T. maculilabris*	*T. striata*
Ala	GCU	0.45	0.51	0.78	0.66	0.45	0.88	0.53	0.92	0.59	0.63	0.49	0.5	0.82	0.69	0.72	0.82	0.86
Ala	GCC	1.9	1.76	1.67	1.77	1.88	1.53	2.01	1.48	1.82	1.63	1.8	1.99	1.92	1.87	1.88	1.64	1.54
Ala	GCA	1.51	1.54	1.51	1.41	1.51	1.5	1.25	1.52	1.51	1.67	1.58	1.44	1.19	1.33	1.26	1.44	1.5
Ala	GCG	0.14	0.19	0.04	0.15	0.17	0.1	0.21	0.09	0.08	0.07	0.13	0.07	0.07	0.11	0.14	0.1	0.1
Cys	UGU	0.59	1.03	0.86	0.86	0.48	0.9	0.73	0.69	0.89	0.59	0.48	0.53	1.11	1.07	0.93	1.07	1.1
Cys	UGC	1.41	0.97	1.14	1.14	1.52	1.1	1.27	1.31	1.11	1.41	1.52	1.47	0.89	0.93	1.07	0.93	0.9
Asp	GAU	0.39	0.54	0.8	0.74	0.48	0.73	0.39	0.58	0.66	0.58	0.4	0.39	0.57	0.72	0.68	0.68	0.76
Asp	GAC	1.61	1.46	1.2	1.26	1.52	1.27	1.61	1.42	1.34	1.42	1.6	1.61	1.43	1.28	1.32	1.32	1.24
Glu	GAA	1.48	1.6	1.67	1.73	1.35	1.59	1.33	1.61	1.58	1.54	1.6	1.48	1.51	1.54	1.51	1.5	1.52
Glu	GAG	0.52	0.4	0.33	0.27	0.65	0.41	0.67	0.39	0.42	0.46	0.4	0.52	0.49	0.46	0.49	0.5	0.48
Phe	UUU	0.82	0.94	1.14	1.26	1.09	1.15	1.15	1.31	1.07	1.14	1.03	0.85	1.26	0.91	1.04	1.16	1.21
Phe	UUC	1.18	1.06	0.86	0.74	0.91	0.85	0.85	0.69	0.93	0.86	0.97	1.15	0.74	1.09	0.96	0.84	0.79
Gly	GGU	0.39	0.41	0.65	0.65	0.53	0.44	0.53	0.65	0.57	0.68	0.39	0.53	0.41	0.54	0.44	0.65	0.82
Gly	GGC	1.37	1.38	1.35	1.3	1.39	1.36	1.68	1.52	1.38	1.19	1.2	1.45	1.65	1.36	1.4	1.34	0.92
Gly	GGA	1.44	1.45	1.49	1.29	1.19	1.53	0.81	1.07	1.15	1.08	1.67	1.06	0.93	1.12	1.23	1.19	1.48
Gly	GGG	0.79	0.75	0.51	0.76	0.89	0.67	0.98	0.76	0.9	1.05	0.74	0.96	1.02	0.98	0.92	0.82	0.78
His	CAU	0.38	0.28	0.62	0.5	0.46	0.68	0.46	0.84	0.55	0.62	0.35	0.29	0.61	0.56	0.68	0.56	0.68
His	CAC	1.62	1.72	1.38	1.5	1.54	1.32	1.54	1.16	1.45	1.38	1.65	1.71	1.39	1.44	1.32	1.44	1.32
Ile	AUU	0.85	1.03	1.21	1.1	0.98	1.08	1.01	1.42	0.94	1.2	0.99	1	1.37	0.9	1.1	1.24	1.27
Ile	AUC	1.15	0.97	0.79	0.9	1.02	0.92	0.99	0.58	1.06	0.8	1.01	1	0.63	1.1	0.9	0.76	0.73
Lys	AAA	1.68	1.71	1.84	1.9	1.77	1.78	1.74	1.72	1.8	1.72	1.75	1.73	1.7	1.59	1.75	1.8	1.98
Lys	AAG	0.32	0.29	0.16	0.1	0.23	0.22	0.26	0.28	0.2	0.28	0.25	0.27	0.3	0.41	0.25	0.2	0.02
Leu2	UUA	0.76	1.13	1.52	1.54	1.3	1.5	1.15	1.71	1.36	1.8	1.01	0.76	1.6	1.1	1.38	1.43	1.65
Leu2	UUG	0.34	0.25	0.29	0.31	0.28	0.31	0.36	0.31	0.25	0.33	0.22	0.21	0.23	0.31	0.25	0.22	0.25

Leu1	CUU	0.69	0.84	1.15	1.1	1.14	1.52	1	1.13	0.85	1.19	0.81	0.81	1.11	0.97	1.03	1.13	1.31
Leu1	CUC	0.9	0.74	0.62	0.71	0.62	0.71	0.83	0.54	0.77	0.49	0.95	0.97	0.88	1.19	1.13	0.96	0.85
Leu1	CUA	2.59	2.5	2.18	2	2.18	1.69	2.17	2.03	2.45	1.82	2.63	2.83	1.89	2.02	1.89	1.97	1.76
Leu1	CUG	0.7	0.53	0.24	0.33	0.48	0.27	0.5	0.29	0.31	0.37	0.38	0.42	0.3	0.41	0.31	0.29	0.19
Met	AUA	1.55	1.62	1.59	1.58	1.47	1.6	1.39	1.65	1.62	1.51	1.59	1.53	1.56	1.43	1.63	1.52	1.67
Met	AUG	0.45	0.38	0.41	0.42	0.53	0.4	0.61	0.35	0.38	0.49	0.41	0.47	0.44	0.57	0.37	0.48	0.33
Asn	AAU	0.49	0.42	0.65	0.62	0.57	0.56	0.35	0.68	0.52	0.8	0.42	0.49	0.64	0.53	0.62	0.57	0.85
Asn	AAC	1.51	1.58	1.35	1.38	1.43	1.44	1.65	1.32	1.48	1.2	1.58	1.51	1.36	1.47	1.38	1.43	1.15
Pro	CCU	0.51	0.3	0.67	0.65	0.54	1.01	0.72	0.97	0.69	0.51	0.35	0.29	0.8	0.78	0.93	0.91	0.85
Pro	CCC	0.83	0.87	0.77	0.97	0.87	0.98	1.16	0.67	0.62	0.61	0.99	0.92	1.13	1.51	1.42	1.14	0.75
Pro	CCA	2.4	2.69	2.44	2.03	2.33	1.88	1.88	2.21	2.54	2.47	2.43	2.53	1.88	1.53	1.38	1.75	2.21
Pro	CCG	0.26	0.14	0.12	0.35	0.27	0.13	0.24	0.14	0.15	0.41	0.23	0.25	0.19	0.18	0.28	0.2	0.2
Gln	CAA	1.68	1.7	1.76	1.73	1.77	1.67	1.71	1.72	1.8	1.78	1.73	1.68	1.67	1.7	1.84	1.67	1.85
Gln	CAG	0.32	0.3	0.24	0.27	0.23	0.33	0.29	0.28	0.2	0.22	0.27	0.32	0.33	0.3	0.16	0.33	0.15
Arg	CGU	0.28	0.29	0.46	0.74	0.63	0.46	0.39	0.34	0.66	0.51	0.51	0.51	0.46	0.56	0.62	0.56	0.78
Arg	CGC	0.39	0.51	0.86	0.51	0.57	0.8	0.78	0.63	0.66	0.4	0.73	0.51	0.86	0.73	0.73	0.56	0.61
Arg	CGA	2.87	2.91	2.4	2.46	2.34	2.46	2.39	2.63	2.41	2.57	2.48	2.69	2.34	2.37	2.31	2.5	2.61
Arg	CGG	0.45	0.29	0.29	0.29	0.46	0.29	0.44	0.4	0.27	0.51	0.28	0.29	0.34	0.34	0.34	0.39	0
Ser2	UCU	0.65	1	1.22	1	0.91	1.31	0.87	0.94	0.97	1.03	0.84	0.74	1.27	1.09	1.24	1.07	1.19
Ser2	UCC	1.86	0.93	1.17	1.21	1.22	1.59	1.58	1.15	1.29	1.05	1.52	1.24	1.61	1.6	1.31	1.61	1.11
Ser2	UCA	2.01	2.77	2.36	2.42	2.49	1.86	2.12	2.63	2.48	2.57	2.24	2.6	1.86	1.91	2.27	2.02	2.32
Ser2	UCG	0.29	0.19	0.2	0.23	0.26	0.2	0.35	0.27	0.09	0.21	0.19	0.21	0.25	0.23	0.14	0.17	0.15
Ser1	AGU	0.12	0.33	0.25	0.35	0.17	0.27	0.07	0.23	0.16	0.47	0.21	0.23	0.25	0.21	0.17	0.34	0.44
Ser1	AGC	1.08	0.79	0.79	0.79	0.96	0.77	1.01	0.78	1.01	0.68	1	0.99	0.75	0.95	0.86	0.78	0.8
Thr	ACU	0.43	0.3	0.56	0.83	0.46	0.74	0.47	0.57	0.73	0.56	0.34	0.35	0.59	0.49	0.51	0.56	0.71
Thr	ACC	1.2	1.34	1	1.23	1.38	1.04	1.55	1.23	1.21	1.21	1.23	1.37	1.53	1.59	1.5	1.45	1.23
Thr	ACA	2.22	2.23	2.35	1.82	2	2.14	1.83	2	1.98	2.16	2.28	2.18	1.77	1.84	1.87	1.89	1.94
Thr	ACG	0.15	0.13	0.09	0.12	0.15	0.08	0.15	0.19	0.08	0.07	0.15	0.11	0.1	0.08	0.12	0.1	0.12
Val	GUU	0.98	1.1	1.33	1.19	1.14	1.22	1.31	1.26	1.13	1.25	1.05	1.03	1.53	0.9	1.15	1.1	1.54

Val	GUC	1.07	0.69	0.63	0.68	0.99	0.93	1.17	0.8	0.56	0.9	1.1	1.07	1.04	1.19	0.91	1.15	0.51
Val	GUA	1.4	1.82	1.74	1.62	1.32	1.41	0.88	1.5	1.81	1.38	1.38	1.36	0.96	1.34	1.35	1.33	1.52
Val	GUG	0.54	0.39	0.3	0.51	0.55	0.43	0.64	0.44	0.49	0.47	0.47	0.54	0.47	0.57	0.59	0.42	0.42
Trp	UGA	1.6	1.61	1.8	1.7	1.55	1.63	1.5	1.65	1.64	1.59	1.7	1.5	1.7	1.56	1.54	1.66	1.79
Trp	UGG	0.4	0.39	0.2	0.3	0.45	0.37	0.5	0.35	0.36	0.41	0.3	0.5	0.3	0.44	0.46	0.34	0.21
Tyr	UAU	0.58	0.93	1.04	0.86	0.89	0.94	0.86	1.22	0.66	0.96	1.02	0.7	1.06	0.74	0.93	1.1	1.19
Tyr	UAC	1.42	1.07	0.96	1.14	1.11	1.06	1.14	0.78	1.34	1.04	0.98	1.3	0.94	1.26	1.07	0.9	0.81

[a]RSCU = observed frequency of a codon / expected frequency assuming equal usage of all synonymous codons for the same amino acid. Values > 1 indicate overrepresented codons, values < 1 indicate underrepresented codons.

Table S5. Climatic type classification of 36 species

Species	Cluster[a]
Acritoscincus duperreyi	1
Ateuchosaurus chinensis	2
Chalcides ocellatus	1
Chalcides sepsoides	1
Cyclodomorphus gerrardii	3
Eulamprus heatwolei	1
Eutropis multifasciata	4
Isopachys gyldenstolpei	3
Liopholis kintorei	1
Lipinia microcerca	3
Mesoscincus schwartzei	3
Mochlus sundevallii	3
Panaspis annettesabinae	3
Plestiodon capito	5
Plestiodon chinensis	2
Plestiodon egregius	3
Plestiodon elegans	2
Plestiodon liui	2
Plestiodon quadrilineatus	2
Plestiodon tunganus	5
Scincella huanrenensis	5
Scincella modesta	2
Scincella reevesii	2
Scincella vandenburghi	2
Sphenomorphus cryptotis	2
Sphenomorphus incognitus	2

Sphenomorphus indicus	2
Sphenomorphus maculatus	3
Spondylurus nitidus	3
Tiliqua gigas gigas	4
Tiliqua occipitalis	1
Tiliqua scincoides	3
Trachylepis maculilabris	2
Trachylepis striata	3
Tropidophorus hainanus	2
Tropidophorus hangnam	3

[a]Cluster values represent the different climate types into which the skink species were classified in this study. Different values correspond to different climate types, related to Figure 2,4A, 5.

Table S7. The best partition schemes and nucleotide substitution models for mitochondrial data using PartitionFinder, where tree1 represents the phylogenetic tree constructed with 116 species and tree2 represents the phylogenetic tree constructed with 38 species, Related to Figure 1,2.

Tree	Subset	Best Model	Subset Partitions
1	1	GTR+I+G	4895-5766\2, 4485-4710\2, 1-408\2, 2451-3198\2, 3199-3814\2
	2	GTR+I+G	3816-4484\2, 2-408\2, 4712-4894\2, 4896-5766\2, 5768-6892\2
	3	GTR+I+G	5767-6892\2, 4711-4894\2, 409-490\2, 3815-4484\2
	4	GTR+I+G	410-490\2, 7207-8199, 8200-8678
	5	SYM+I+G	491-1478\2
	6	GTR+I+G	1480-1928\2, 492-1478\2
	7	GTR+I+G	1479-1928\2, 1929-2450\2
	8	GTR+I+G	4486-4710\2, 3200-3814\2, 2452-3198\2, 1930-2450\2
	9	TVM+G	6893-7206\2
	10	TVM+I+G	6894-7206\2
2	1	K81UF+I+G	1-680
	2	TVM+I+G	681-844, 5954-6984
	3	TRNEF+I+G	845-2389\3
	4	HKY+I+G	846-2389\3
	5	TRN+I+G	847-2389\3
	6	SYM+I+G	2390-3076\3, 3077-3859\3
	7	GTR+I+G	4992-5953\3, 3861-4990\3, 2391-3076\3, 3078-3859\3
	8	TIM+I+G	2392-3076\3, 3079-3859\3, 3862-4990\3, 6987-7329\3
	9	GTR+I+G	7330-7623\3, 9004-10815\3, 3860-4990\3, 7624-9003\3, 4991-5953\3, 6985-7329\3
	10	TIM+I+G	4993-5953\3, 9006-10815\3, 7626-9003\3
	11	TVM+I+G	7331-7623\3, 9005-10815\3, 7625-9003\3, 6986-7329\3
	12	K81UF+I+G	7332-7623\3
	13	TVM+G	10816-11337\3
	14	HKY+I+G	10817-11337\3
	15	HKY+I+G	10818-11337\3

Table S10. Variance inflation factor (VIF)[a] values for bioclimatic variables included in the multicollinearity assessment, related to Figure 4A,4B.

variable	VIF
bio15	NA
bio18	5.653414
bio19	1.323391
bio2	2.73579
bio4	1.660953
bio5	2.59317
bio8	4.033178

[a]VIF measures multicollinearity in regression analysis. Values > 5 indicate substantial collinearity.
